# Ets1-regulated endothelial-secreted factors promote compact myocardial growth and contribute to the pathogenesis of ventricular non-compaction

**DOI:** 10.1093/cvr/cvaf264

**Published:** 2026-02-24

**Authors:** Lu Wang, Zeyu Chen, Aiden Tang, Zhe Yu, Bin Zhou, Sylvia M. Evans, Ju Chen, Paul Grossfeld

**Affiliations:** 1 Department of Pediatrics, UCSD School of Medicine, La Jolla, CA 92093, USA; 2 Department of Medicine, University of California San Diego, La Jolla, CA 92093, USA; 3 Department of Pharmacology, Skaggs School of Pharmacy and Pharmaceutical Sciences, University of California San Diego, La Jolla, CA 92093, USA; 4 Department of Pediatrics, The University of Chicago, Chicago, IL 60637, USA; 5 Division of Cardiology, Rady Children’s Hospital, San Diego, CA 92123, USA.

**Keywords:** ventricular non-compaction, cardiomyocyte proliferation, endothelial-secreted factor, extracellular matrix, Notch1 signaling

## Abstract

**Aims:**

Thinning of the compact myocardium is a major contributor to adverse outcomes in ventricular non-compaction, the third most common form of cardiomyopathy. Endothelial-specific deletion of *Ets1*, a gene associated with Jacobsen syndrome, causes ventricular non-compaction with reduced compact myocardium. However, the mechanisms by which pathological cardiac endothelium impairs compact myocardium growth remain poorly understood.

**Methods and results:**

To uncover the mechanisms underlying compact myocardium thinning and identify therapeutic endothelial-secreted factors, we performed single-cell RNA sequencing. Aberrant cardiomyocyte and endothelial cell states were observed in non-compacted ventricles. Conditional deletion of *Ets1* in either the endocardium or coronary endothelium impaired compact myocardial growth. In endocardium, *Ets1* deficiency suppressed Notch1 signaling by upregulating *Dlk1* and downregulating *Dll4*, both direct Ets1 targets. In coronary endothelium, *Ets1* deficiency reduced the expression of its direct targets *Hmcn1*, *Slit2*, and *Col18a1*, three extracellular matrix (ECM) components that promote compact myocardial proliferation. Notably, treatment with these ECM proteins or the Notch1 effector Nrg1 restored the impaired compact myocardial proliferation.

**Conclusions:**

These findings highlight Ets1-regulated endothelial-secreted factors as essential for compact myocardium development and suggest novel therapeutic targets for ventricular non-compaction.

## Graphical Abstract

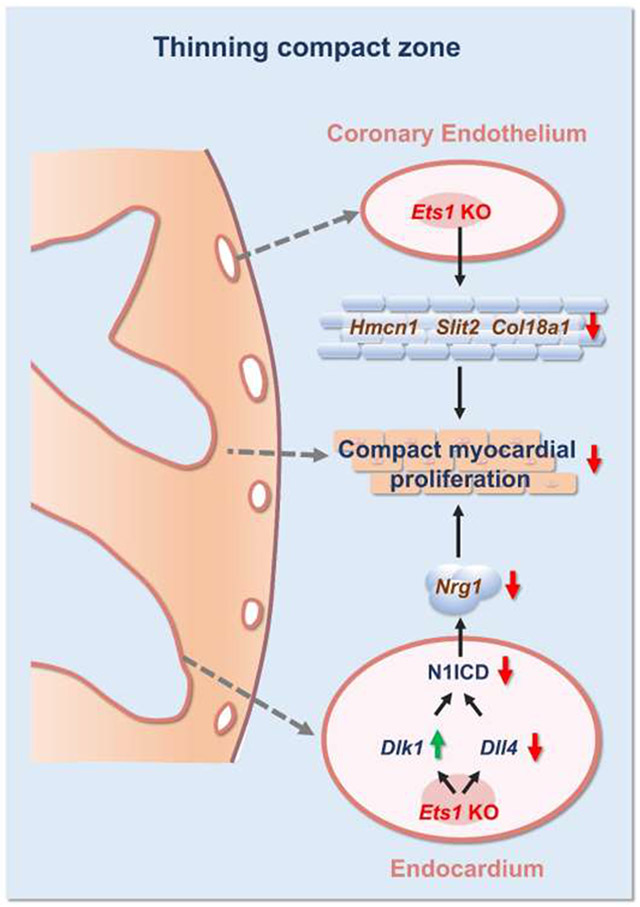

## 1. Introduction

Ventricular non-compaction is the third most common form of cardiomyopathy and is characterized by prominent myocardial trabeculations and a thinned compact zone.^1,2^ The trabecular layer of ventricular myocardium is a sponge-like network of cardiomyocytes, playing a crucial role in development of the ventricular chamber^3^. In healthy individuals, increased trabeculation occurs as a physiological adaptation to altered loading conditions, such as athletic training, and is commonly observed in healthy African Americans.^4,5^ In the absence of inherited cardiac conditions or related symptoms, excessive trabeculation is generally considered benign.^5,6^ However, increasing evidence suggests that a thinner compact myocardium may be an independent risk factor for adverse clinical outcomes in ventricular non-compaction.^1,7,8^ Therefore, investigating mechanisms underlying the thinning of compact zone myocardium in ventricular non-compaction will contribute to development of strategies to address the pathological nature of this condition.

Compact zone cardiomyocyte proliferation drives progressive thickening of the ventricular wall. This process involves proliferation of the compact zone myocardium, and its expansion during late embryonic stages, which leads to incorporation of the trabecular myocardium.^9,10^ Defects in compact cardiomyocyte proliferation lead to thinning of compact zone myocardium in ventricular non-compaction. Genome-wide association studies in humans have shown that non-compaction cardiomyopathy is a genetically driven condition resulting from mutations in genes encoding sarcomeric, cytoskeletal, and nuclear membrane proteins.^11,12^ Additionally, recent research has identified dysfunction in the coronary endothelium and endocardium as contributing factors to the development of non-compaction cardiomyopathy.^13-16^ However, the underlying mechanisms of the disease and potential therapeutic strategies remain poorly understood.

Ventricular non-compaction has been observed in Jacobsen syndrome (JBS, OMIM #147791), a chromosomal disorder caused by deletions in distal 11q that leads to multiple developmental defects, including congenital heart defects (CHDs).^17^ Among the genes affected, *Ets1* deficiency has been associated with CHDs in JBS.^18,19^ In our previous study, we showed that endothelial-specific deletion of *Ets1* in mice leads to a ventricular non-compaction phenotype, characterized by thinning of compact myocardium and overgrowth of trabecular layer.^19^ We found that coronary endothelial deletion of *Ets1* impaired coronary vascular development by reducing the expression of six key angiogenesis-related genes, while endocardial deletion of *Ets1* increased trabecular cardiomyocyte proliferation through upregulation of Tgfb2/Tgfb1/Smad2 signaling. However, the molecular mechanism by which *Ets1*-deficient cardiac endothelium impairs the growth of the compact zone myocardium remains unknown. Thus, using the *Ets1* endothelial knockout model that mimics human ventricular non-compaction, here we further investigate the contribution of the coronary endothelium and/or endocardium to the thinning of the compact zone myocardium and the underlying molecular mechanisms. By understanding how the cardiac endothelium regulates compact myocardial growth, we aim to facilitate the development of targeted therapies for ventricular non-compaction.

In this study, using single-cell RNA sequencing (scRNA-seq), we revealed transcriptomic and cellular state alterations in cardiomyocytes and cardiac endothelial cells in ventricular non-compaction caused by *Ets1* endothelial conditional knockout (eKO), and identified key cell types involved in the pathogenesis of ventricular non-compaction. We also showed that *Ets1* deficiency in either endocardium or coronary endothelium led to thinning of compact zone myocardium and elucidated the underlying molecular mechanisms. Additionally, we demonstrated a restorative effect of Hmcn1, Slit2, and Col18a1, three identified extracellular matrix (ECM) components, as well as the Notch1 downstream effector Nrg1, on the reduced proliferation of compact zone cardiomyocytes caused by *Ets1* eKO. Uncovering how cardiac endothelium regulates compact myocardial growth will contribute to the development of targeted therapies for ventricular non-compaction and potentially other disease processes involving cardiomyocyte proliferation.

## 2. Methods

Comprehensive methods and relevant references are included in the Supplemental Material. Information on antibodies, probes and primers can be found in Tables S2 and S3.

### 2.1 Mice

All animal experiments were conducted in compliance with the National Institutes of Health’s *Guide for the Care and Use of Laboratory Animals*^20^ and were approved by the Institutional Animal Care and Use Committee at the University of California, San Diego. *Ets1* conditional knockout mice were generated by breeding *Ets1* flox/flox (fl/fl) mice with *Tie2*Cre (Jackson Laboratory, #008863), *Pdgfb*CreERT2 (provided by Dr. Marcus Fruttiger, University College London) and *Nfatc1*Cre (provided by Dr. Bin Zhou, The University of Chicago) mice. Cardiomyocyte-specific FUCCI-expressing mice were generated by breeding R26FUCCI2aR mice (provided by Dr. Ian James Jackson, University of Edinburgh) with *Xmlc2*Cre mice.^21^ Embryonic mice of both sexes at various developmental stages were used without prior genotyping for gender. All animals were maintained under the care of the UCSD Animal Care Program, and all experimental procedures were approved by the UCSD Institutional Animal Care and Use Committee. *Ets1* fl/fl, *Tie2*Cre, *Pdgfb*CreERT2 and *Nfatc1*Cre mice were maintained on a C57BL/6 background, while R26FUCCI2aR and *Xmlc2*Cre were maintained on an FVB/N background. Genotyping was performed using primers listed in Tables S3. Mice were anesthetized with ketamine (150 mg/kg) and xylazine (15 mg/kg) via intraperitoneal injection before tissue collection. At the experimental endpoint, mice were euthanized by CO_2_ inhalation in accordance with institutional guidelines.

### 2.2 Statistical Analysis

Statistical analyses were performed using GraphPad Prism 8.4.3. Data normality was assessed using the Kolmogorov-Smirnov, Shapiro-Wilk, and D’Agostino-Pearson normality tests. Results are presented as mean ± SEM. For normally distributed data, an unpaired two-tailed Student’s *t*-test was used for comparisons between two groups, while one-way ANOVA followed by Tukey's multiple comparisons test was applied for comparisons among more than two groups. For non-normally distributed data or when *n* < 6, a nonparametric test was used for two-group comparisons. No experiment-wide/across-test multiple test correction was applied. When multiple comparisons were conducted, multiplicity-adjusted *P*-values were reported; otherwise, raw *P*-values were reported. A value of *P* < 0.05 was considered statistically significant. Biological replicates were used, with *n* representing the number of mice.

## 3. Results

### 3.1 Alterations in cardiomyocyte states in ventricular non-compaction caused by *Ets1* endothelial deletion.

**Figure 1.**
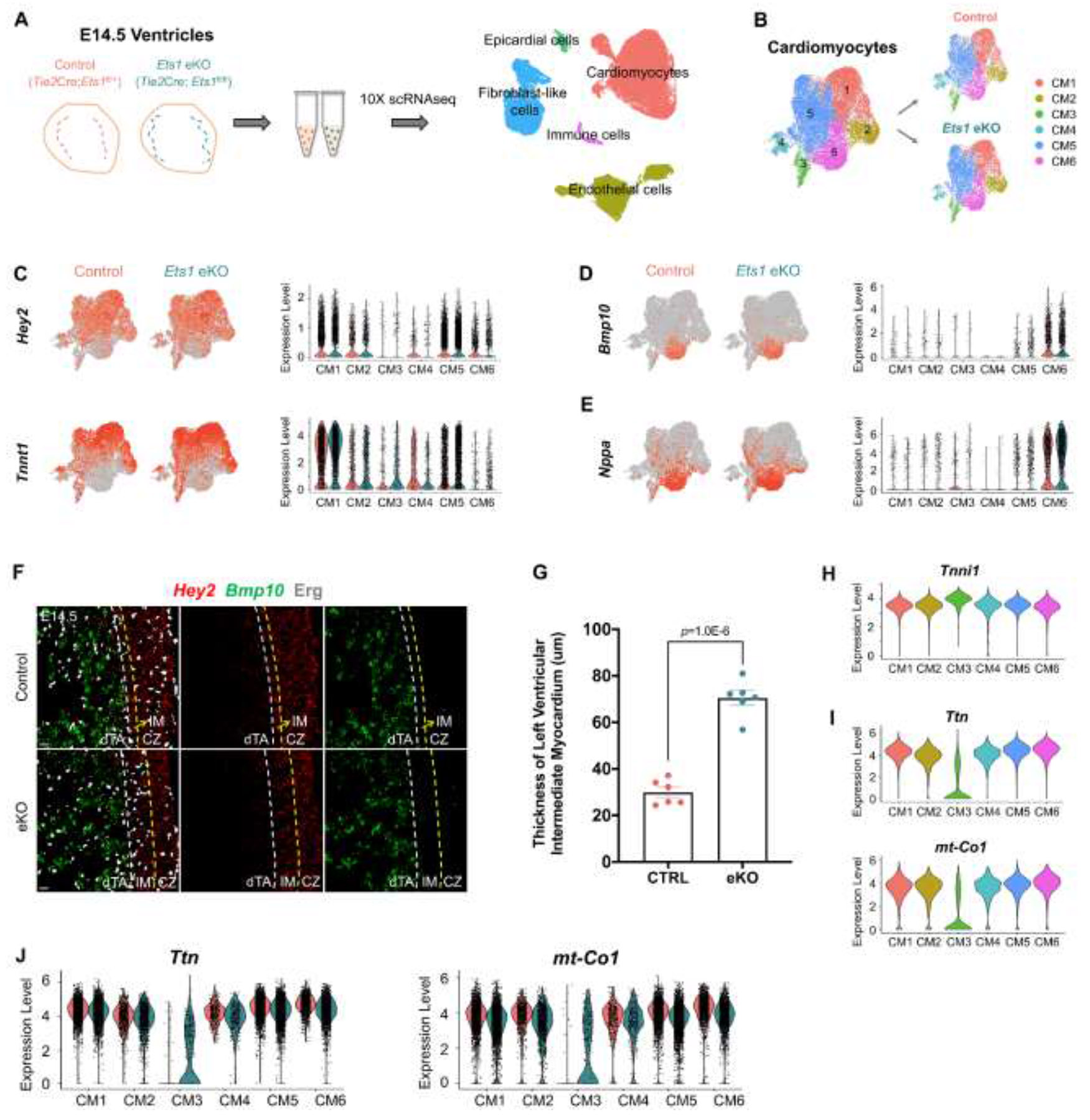
Alterations in cardiomyocyte states in ventricular non-compaction caused by *Ets1* endothelial deletion. (A) Schematic of single-cell RNA sequencing (scRNA-seq) performed on E14.5 *Ets1* endothelial conditional knockout (eKO) and control ventricles, along with a UMAP representation of five distinct cell populations identified. (B) UMAP visualization of the cardiomyocyte (CM) population. (C-E) Feature and violin plots showing the expression of (C) *Hey2* and *Tnnt1*, (D) *Bmp10*, and (E) *Nppa*. (F) Representative confocal images of RNAscope *in situ* hybridization (ISH) for *Hey2*, a compact zone (CZ) myocardium marker, and *Bmp10*, a trabecular layer (TA) myocardium marker, along with immunofluorescence staining for Erg, which lines the trabecular layer, to show CZ, distal trabecular layer (dTA), and intermediate myocardium (IM) in control and *Ets1* eKO mice at E14.5. The yellow dashed line distinguishes CZ from TA, while the white dashed line separates IM from dTA. Scale bars: 20 μm. (G) Graph showing IM thickness of left ventricles in control and eKO mice at E15.5 (CTRL, n=6; eKO, n=6). Data are presented as mean ± SEM and *P* value was calculated using an unpaired two-tailed Student’s t-test. (H and I) Violin plots showing the expression of (H) *Tnni1*, (I) *Ttn* and *mt-Co1*. J, Violin plots showing the expression of *Ttn* and *mt-Co1*.

To identify key cell types involved in the pathogenesis of ventricular non-compaction and to uncover the molecular mechanisms underlying the thinning of compact zone myocardium, we performed scRNA-seq on E14.5 *Ets1* eKO (*Tie2*Cre;*Ets1*^fl/fl^) and control (*Tie2*Cre;*Ets1*^fl/+^) ventricles, as we have previously demonstrated that *Ets1* eKO results in a ventricular non-compaction phenotype.^19^ Unsupervised clustering identified five distinct cell populations: cardiomyocytes (CMs), endothelial cells (ECs), fibroblast-like cells, epicardial cells, and immune cells (Figure 1A). The top eight genes used to define each cell population is presented in Figure S1A. CMs were identified by markers including *Tnnt2*, *Tnnc1*, *Actc1*, *Tnni1*, *Tpm1*, *Tnni3*, *Nexn* and *Ttn*, while ECs were identified by markers including *Pecam1*, *Ecscr*, *Egfl7*, *Flt1*, *Cd93*, *Cdh5*, *Emcn* and *Ctla2a* (Figure S1A).

As the ventricular non-compaction phenotype caused by endothelial deletion of *Ets1* is primarily reflected in the myocardium, we first analyzed the scRNA-seq data of cardiomyocytes to further investigate changes in the cardiomyocyte transcriptome underlying ventricular non-compaction. Sub-clustering of the cardiomyocyte cluster identified six distinct subpopulations (CM1-CM6) (Figure 1B). The top genes defining each CM subpopulation are shown in Figure S1B. CM1 expressed compact zone cardiomyocyte marker genes, such as *Hey2* and *Tnnt1* (Figure 1C), while CM6 expressed trabecular cardiomyocyte marker genes, including *Bmp10* and *Nppa* (Figure 1D and 1E). CM2 and CM5 were not distinguished by compact zone and trabecular cardiomyocyte marker genes, but were characterized by high expression of proliferation-associated genes, such as *Top2a* and *Mki67* (Figure S1C). CM4 was identified as atrioventricular canal cardiomyocytes, marked by *Irx3*, *Tbx3*, and *Rspo3* (Figure S1D).

Unlike the expression pattern of *Bmp10* in cardiomyocytes, *Nppa* was also highly expressed in a subset of CMs in CM2 and CM5, in addition to CM6 (Figure 1D and 1E). These cardiomyocytes correspond to the intermediate myocardium described in previous studies.^10,15^ Moreover, compared to controls, *Ets1* eKO ventricles exhibited a greater number of *Nppa*^+^ cardiomyocytes in CM2 and CM5 (Figure 1E), indicating an increased presence of intermediate myocardium cardiomyocytes. To further confirm the presence of a larger intermediate myocardium region in *Ets1* eKO ventricles, Erg immunofluorescence was used to label the endocardium to distinguish the compact zone from the trabecular layer, and RNAscope *in situ* hybridization (ISH) was performed to visualize the expression of *Hey2* and *Bmp10*. The ventricular wall was divided into *Hey2*^hi^ (high expression of *Hey2*) and *Bmp10*^−^ compact zone, *Hey2*^hi^ and *Bmp10*^−^ / *Bmp10*^lo^ (low expression of *Bmp10*) proximal trabecular layer, also known as intermediate myocardium, and *Hey2*^−^ / *Hey2*^lo^ and *Bmp10*^hi^ distal trabecular layer (Figure 1F). Compared to controls, in addition to the previously observed thinner compact zone, we also found that the intermediate myocardium in *Ets1* eKO ventricles was obviously thicker, indicating a defect in the compaction of the proximal trabecular layer caused by *Ets1* eKO (Figure 1F and 1G). It has been reported that compact zone myocardial expansion drives trabecular meshwork incorporation into the compact zone myocardium.^10^ Since we previously demonstrated that *Ets1* eKO leads to reduced compact zone cardiomyocyte proliferation,^19^ our current findings, together with the established model, suggest that reduced cardiomyocyte proliferation decreased compact zone expansion, thereby impairing proximal trabecular layer compaction.

Furthermore, we identified a population of immature cardiomyocytes, labeled as CM3. These cells exhibited high expression of immature cardiomyocyte marker genes, such as *Tnni1* (Figure 1H; Figure S1E), but low expression of mature cardiomyocyte marker genes, including *Ttn* and *mt-Co1* (Figure 1I; Figure S1E).^13,22,23^ Notably, compared to controls, expression levels of mature cardiomyocyte marker genes *Ttn* and *mt-Co1* were significantly increased in CM3 of *Ets1* eKO ventricles (Figure 1J). This suggests the presence of a population of immature cardiomyocytes undergoing early-onset maturation in ventricular non-compaction caused by endothelial deletion of *Ets1*, further supporting the association between ventricular non-compaction and premature cardiomyocyte maturation.^13^ Overall, the results above suggest that endothelial loss of *Ets1* altered the transcriptome and cell states of cardiomyocytes in ventricular non-compaction.

### 3.2 Transcriptomic alterations in cardiac endothelial cells in ventricular non-compaction caused by *Ets1* endothelial deletion.

**Figure 2.**
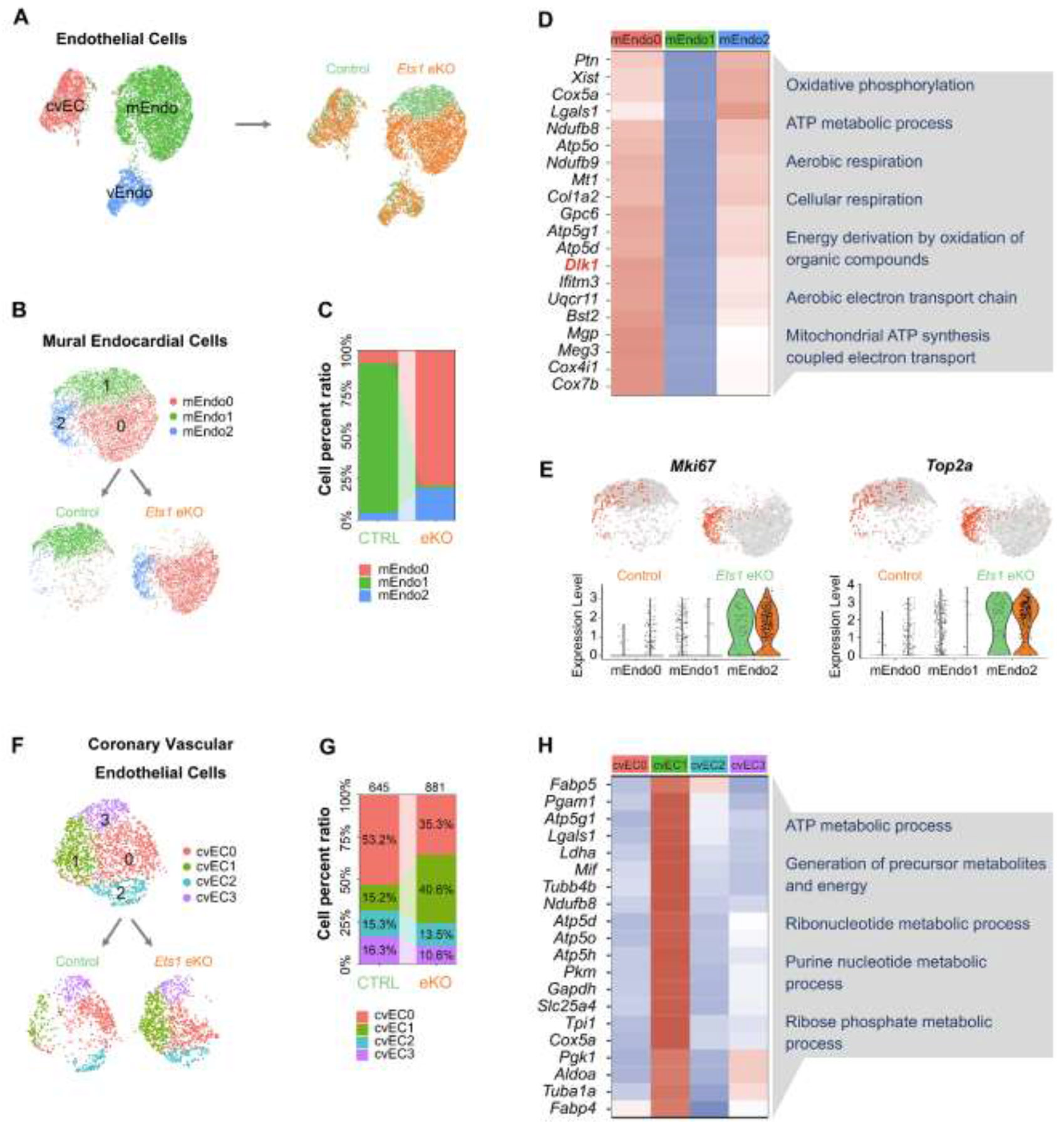
Transcriptomic alterations in cardiac endothelial cells in ventricular non-compaction caused by *Ets1* endothelial deletion. (A) UMAP visualization of the three cardiac endothelial cell (EC) subpopulations (left panel) and the merged EC distribution from *Ets1* endothelial conditional knockout (eKO) and control ventricles (right panel). Green dots represent ECs from control ventricles, while orange dots represent ECs from *Ets1* eKO ventricles. (B) UMAP visualization of the mural endocardial cell (mEndo) population. (C) Proportional distribution of the three mEndo subpopulations in *Ets1* eKO and control ventricles. (D) Gene ontology (GO) enrichment analysis of mEndo0-specific marker genes. (E) Feature and violin plots displaying the expression of genes specific to proliferating cells. (F) UMAP visualization of the coronary vascular endothelial cell (cvEC) population. (G) Proportional distribution of the four cvEC subpopulations in *Ets1* eKO and control ventricles. The total numbers of cvECs for each group (control and eKO), along with the percentage contribution of each sub-cluster to the total cvEC pool in the respective group, are shown. (H) GO enrichment analysis of cvEC1-specific marker genes.

Building on our previous finding that *Ets1*-deficient cardiac endothelium plays a central role in driving ventricular non-compaction, we next investigated the transcriptomic alterations in cardiac endothelial cells associated with this condition. Unsupervised clustering of the EC population identified three subpopulations (Figure 2A, left panel): coronary vascular endothelial cells (cvECs; *Fabp4*^+^) (Figure S2A), mural endocardial cells (mEndos; *Npr3*^hi^) (Figure S2B) and valve endocardial cells (vEndos; *Nfatc1*^+^ and *Prox1*^+^, and/or *Nfatc1*^+^ and *Hey2*^+^) (Figure S2C). Notably, the transcriptome of EC clusters in *Ets1* eKO and control ventricles exhibited significant differences (Figure 2A, right panel). To specifically compare *Ets1* eKO and control ventricle ECs, we further sub-clustered the two cardiac endothelial populations that potentially influence compact myocardium: mural endocardial and coronary vascular endothelial cells. Sub-clustering of the mural endocardial cell cluster revealed three distinct subpopulations, labeled as mEndo0, mEndo1, and mEndo2 (Figure 2B). Among them, the mEndo1 cluster primarily consisted of control mural endocardial cells, whereas mEndo0 and mEndo2 clusters were predominantly composed of *Ets1* eKO mural endocardial cells (Figure 2B and 2C). The expression of the top genes defining each mural endocardial cell subpopulation are shown in Figure S3A. Gene Ontology (GO) enrichment analysis of mEndo0-specific marker genes identified oxidative phosphorylation, ATP metabolic process, aerobic respiration, and other top biological processes (Figure 2D), and these mEndo0-specific marker genes were also highly expressed in mEndo2, indicating altered energy metabolism in mural endocardial cells of *Ets1* eKO ventricles with ventricular non-compaction. Further analysis revealed that one of the two distinct *Ets1* eKO mural endocardial cell subpopulations, specifically the mEndo2 cluster, was composed of mural endocardial cells with high expression of genes associated with proliferation, such as *Top2a* and *Mki67* (Figure 2E; Figure S3A). This suggests the presence of a specific population of proliferative mural endocardial cells in the non-compacted ventricles resulting from *Ets1* eKO.

Sub-clustering of the coronary vascular EC cluster revealed four distinct subpopulations, labeled as cvEC0, cvEC1, cvEC2 and cvEC3 (Figure 2F). The top genes defining each coronary vascular EC subpopulation are shown in Figure S3B. CvEC0 and cvEC1 clusters were characterized by expression of capillary EC marker genes such as *Rgcc* (Figure S4A); cvEC2 cluster was defined by venous EC marker genes such as *Nr2f2* and *Dab2* (Figure S4B); and cvEC3 cluster was defined by arterial EC marker genes such as *Gja5* and *Gja4* (Figure S4C). The total proportion of cvEC0 and cvEC1 cells among all coronary vascular ECs was increased in *Ets1* eKO ventricles compared to controls, whereas both cvEC2 and cvEC3 cells showed a reduction in proportion (Figure 2F and 2G). These findings indicate impaired coronary artery and vein development in *Ets1* eKO ventricles with ventricular non-compaction, supporting our previous observations in *Ets1* eKO mice, where the coronary vasculature appears truncated with decreased arborization. Notably, the proportion of cvEC1 cells among all coronary vascular ECs was significantly increased in *Ets1* eKO ventricles compared to controls (Figure 2F and 2G). GO enrichment analysis of cvEC1-specific marker genes identified the top biological processes as ATP metabolic process, generation of precursor metabolites and energy, and others (Figure 2H), indicating altered energy metabolism in coronary endothelial cells of *Ets1* eKO ventricles. Together, these data demonstrate that transcriptomes and states of endocardial and coronary endothelial cells are significantly altered in *Ets1* eKO ventricles with ventricular non-compaction, implying that both *Ets1*-deficient cardiac endothelial populations may contribute to thinning of compact myocardium.

### 3.3 *Ets1* deficiency in either endocardium or coronary endothelium contributes to thinning of compact myocardium.

Next, we set out to examine whether loss of *Ets1* in either coronary endothelium or endocardium could individually lead to thinning of the compact myocardium. Conditional knockout mouse lines were used to delete *Ets1* in either of these two cardiac endothelial lineages. Specifically, the *Pdgfb*CreERT2 driver line was used to delete *Ets1* in coronary vascular endothelial cells, while the *Nfatc1*Cre driver line was used to delete *Ets1* in endocardial cells.

**Figure 3.**
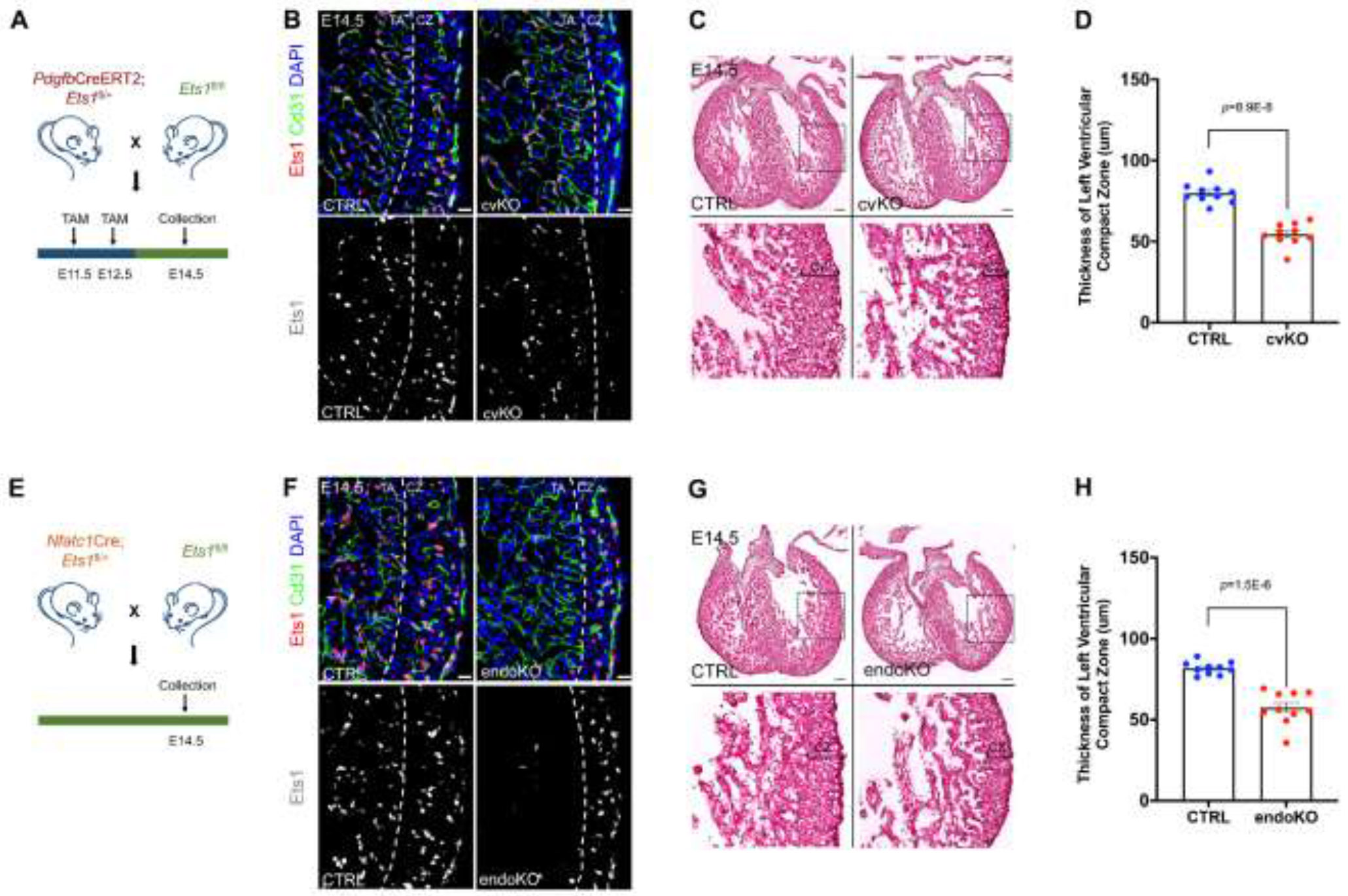
Loss of *Ets1* in either coronary endothelium or endocardium can lead to the thinning of the compact myocardium. (A) Schematic illustrating the strategy for coronary vascular endothelial cell-specific loss of *Ets1* using the *Pdgfb*CreERT2 driver. (B) Ets1 immunofluorescence showing that Ets1 protein was undetectable in coronary vascular endothelial cells of *Ets1* coronary vascular endothelial conditional knockout (cvKO) embryonic hearts at E14.5. Scale bars: 20 μm. (C) Representative images of heart sections stained with Hematoxylin and Eosin, showing compact myocardium thickness in control and *Ets1* cvKO mice at E14.5. Scale bars: 100 μm. (D) Graph showing the compact myocardium thickness of left ventricles in control and *Ets1* cvKO mice at E14.5 (CTRL, n=10; cvKO, n=10). (E) Schematic illustrating the strategy for endocardial-specific loss of *Ets1* using the *Nfatc1*Cre driver. (F) Ets1 immunofluorescence showing that Ets1 protein was undetectable in endocardial cells of *Ets1* endocardial conditional knockout (endoKO) embryonic hearts at E14.5. Scale bars: 20 μm. (G) Representative images of heart sections stained with Hematoxylin and Eosin, showing compact myocardium thickness in control and *Ets1* endoKO mice at E14.5. Scale bars: 100 μm. (H) Graph showing the compact myocardium thickness of left ventricles in control and *Ets1* endoKO mice at E14.5 (CTRL, n=10; endoKO, n=10). CZ, compact zone; TA, trabecular layer. For (D) and (H), data are presented as mean ± SEM and *P* values were calculated using an unpaired two-tailed Student’s t-test.

When using the *Pdgfb*CreERT2 driver to delete *Ets1* in coronary vascular ECs, pregnant female mice were given 4-Hydroxytamoxifen by oral gavage at E11.5 and E12.5, and embryonic hearts were collected at E14.5 (Figure 3A). Compared to controls (*Pdgfb*CreERT2;*Ets1*^fl/+^), Ets1 immunofluorescence showed undetectable Ets1 protein in coronary vascular ECs in coronary vascular endothelial conditional knockout (cvKO, *Pdgfb*CreERT2;*Ets1*^fl/fl^) embryonic hearts at E14.5, that was still present in endocardial cells (Figure 3B). Hematoxylin and Eosin (H&E) staining showed that deletion of *Ets1* in coronary vascular ECs resulted in decreased thickness of the compact zone (Figure 3C and 3D). In addition, Cd31 immunofluorescence also revealed a decreased thickness of the compact zone in cvKO mice (Figure 3B). When using the *Nfatc1*Cre driver to delete *Ets1* in endocardial cells, embryonic hearts were also collected at E14.5 (Figure 3E). Compared to controls (*Nfatc1*Cre;*Ets1*^fl/+^), Ets1 immunofluorescence showed undetectable Ets1 protein in endocardial cells in endocardial conditional knockout (endoKO, *Nfatc1*Cre;*Ets1*^fl/fl^) embryonic hearts at E14.5, but Ets1 was still present in coronary vascular ECs (Figure 3F). H&E staining showed that deletion of *Ets1* in endocardial cells also resulted in decreased thickness of the compact zone (Figure 3G and 3H). Furthermore, Cd31 immunofluorescence also revealed a decreased thickness of the compact zone in endoKO mice (Figure 3F).

In our previous study, we found that the thickness of the compact zone was reduced by approximately 50% in *Ets1* eKO mice, where *Ets1* was deleted in both the coronary endothelium and the endocardium.^19^ Here, we observed a decrease of about 30% in the thickness of the compact zone in either cvKO or endoKO mice (Figure 3D and 3H). As we have previously demonstrated that the loss of *Ets1* in cardiac endothelium does not affect compact zone cardiomyocyte apoptosis, and that the thinned compact zone observed in *Ets1* eKO mice is caused by decreased compact zone cardiomyocyte proliferation,^19^ these data together show that *Ets1* deficiency in either endocardium or coronary endothelium can lead to thinning of the compact myocardium by impairing compact zone cardiomyocyte proliferation.

### 3.4 Loss of *Ets1* in the endocardium suppresses the Notch1 signaling pathway, leading to thinning of the compact myocardium.

**Figure 4.**
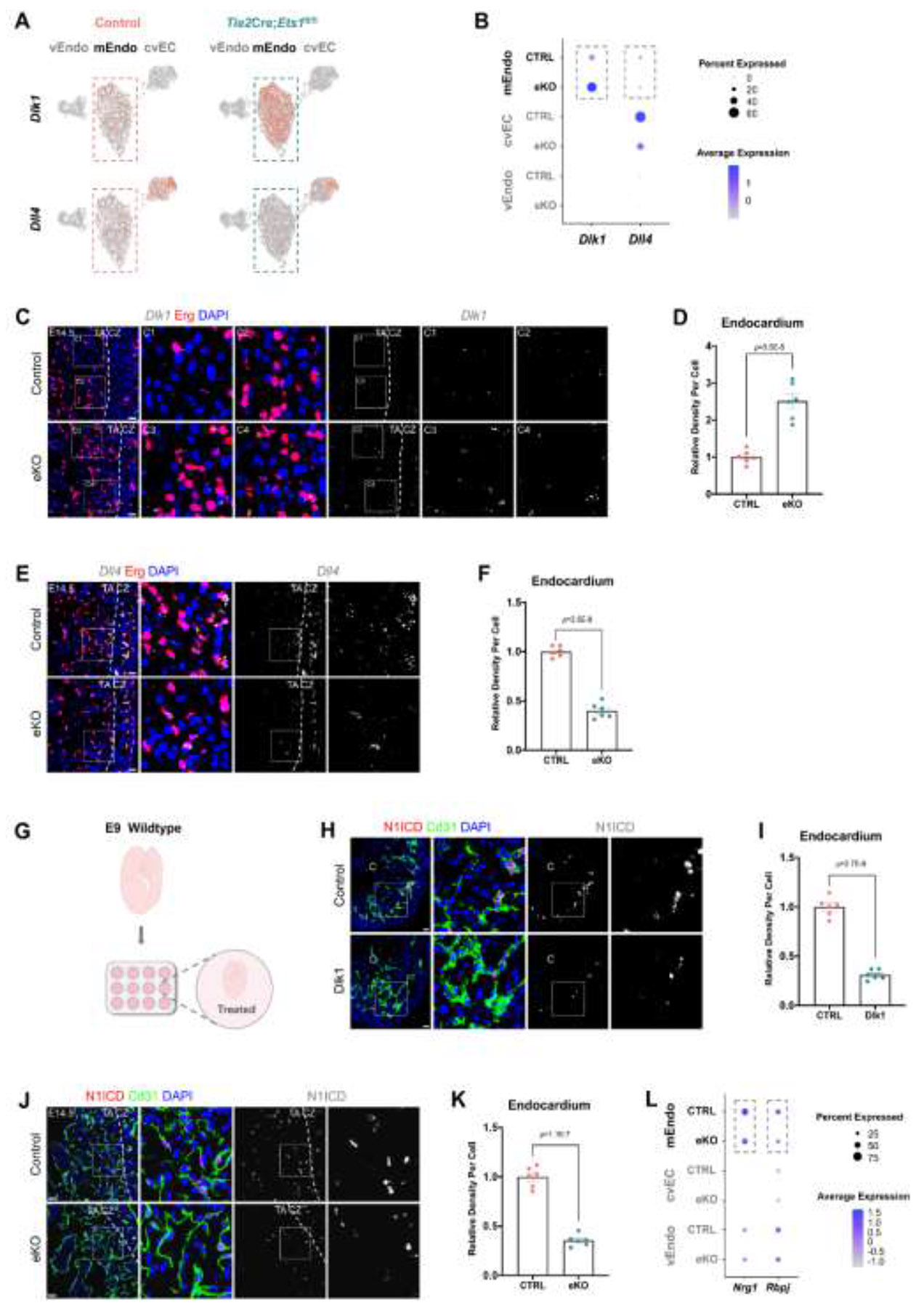
Loss of *Ets1* in the endocardium suppresses the Notch1 signaling pathway. (A and B) (A) Feature and (B) dot plots showing the expression of *Dlk1* and *Dll4* in control and *Ets1* endothelial conditional knockout (eKO) mice. (C-F) Representative confocal images of RNAscope *in situ* hybridization (ISH) for (C) *Dlk1* and (E) *Dll4*, and quantification graphs of signal intensity for (D) *Dlk1* and (F) *Dll4* in the endocardium, showing the expression levels in control and *Ets1* eKO mouse hearts at E14.5 (Control, n=6; eKO, n=6, respectively). Scale bars: 20 μm. (G) Schematic illustrating the culture of E9 mouse embryos. (H and I) (H) Representative confocal images of Notch1 intracellular domain (N1ICD) immunofluorescence and (I) quantification graph of signal intensity for N1ICD in the endocardium of control and Dlk1-treated embryos (Control, n=6; Dlk1, n=6). C, chamber. Scale bars: 20 μm. (J and K) (J) Representative confocal images of N1ICD immunofluorescence and (K) quantification graph of signal intensity for N1ICD in the endocardium, showing the expression levels in control and *Ets1* eKO mouse hearts at E14.5 (Control, n=6; eKO, n=6). Scale bars: 20 μm. (L) Dot plot showing the expression of *Nrg1* and *Rbpj*. cvEC, coronary vascular endothelial cell; mEndo, mural endocardial cell; vEndo, valve endocardial cells; CZ, compact zone; TA, trabecular layer. For (D), (F), (I) and (K), data are presented as mean ± SEM and *P* values were calculated using an unpaired twotailed Student’s t-test.

Next, we investigated the molecular mechanisms underlying the thinning of compact myocardium and began identifying endothelial-secreted factors with potential therapeutic relevance. We first analyzed the scRNA-seq data of the endocardial cell cluster and focused on the two abnormal endocardial cell subpopulations specific to the *Ets1* eKO ventricle, whose transcriptomes were significantly altered. We found that one of the top genes defining the two *Ets1* eKO-specific mEndo subpopulations, mEndo0 and mEndo2, is *Dlk1*, which encodes a ligand for Notch1 (Figure 2D).^24^ Further analysis revealed that *Dlk1* expression in endocardium of *Ets1* eKO mice is significantly increased compared to controls (Figure 4A and 4B; Figure S5A). In addition, we found that *Dll4*, which encodes Dll4, another ligand for Notch1, had decreased expression in endocardium of *Ets1* eKO mice compared to controls (Figure 4A and 4B; Figure S5A). To further investigate expression levels of these two genes in endocardium, RNAscope ISH was performed at E14.5. We found that *Dll4* was more highly expressed in endocardium adjacent to compact myocardium compared to regions farther away (Figure 4E, upper panels), whereas *Dlk1* showed no significant difference in expression between endocardium near and farther from compact myocardium (Figure 4C, upper panels). We also found that *Dlk1* mRNA levels were increased, whereas *Dll4* mRNA levels were decreased in endocardium of *Ets1* eKO mice compared to littermate controls (Figure 4C through 4F).

Dll4 has been reported as a key ligand for Notch1 in endocardium and activates the Notch1 signaling pathway.^9^ However, the role of Dlk1 in Notch1 signaling remains uncertain. Some studies indicate that Dlk1 acts as an Notch1 inhibitor,^24,25^ while others suggest the opposite.^26^ To verify the effect of Dlk1 on Notch1 activation in endocardium, E9 mouse embryos were cultured with or without recombinant Dlk1 protein for 24 hours (Figure 4G) and Notch1 intracellular domain (N1ICD) immunofluorescence was performed to examine Notch1 activation. Compared to controls, Dlk1 treatment reduced N1ICD expression in endocardium, indicating that Dlk1 functions as an inhibitor of Notch1 signaling (Figure 4H and 4I).

Next, we investigated expression levels of N1ICD in endocardium in *Ets1* eKO mice at E14.5, and found that levels of N1ICD in endocardium in *Ets1* eKO mice were significantly decreased compared to littermate controls (Figure 4J and 4K). Ets1 inhibits Dlk1 transcriptional activity by binding to the CpG site 11.^27^ In addition, a combined analysis of published Ets1 chromatin immunoprecipitation sequencing (ChIP-seq) dataset with available RNAPII, H3K4me3, and H3K27ac ChIP-seq data^28^ revealed that Ets1 ChIP peaks were present in the promoter region of *Dll4*, indicating that *Dll4* is also directly regulated by Ets1 (Figure S5B). We further examined mRNA levels of *Dlk1* and *Dll4*, as well as protein levels of N1ICD in endocardium of *Ets1* eKO mice at an early embryonic stage, before the thinning of the compact zone becomes apparent. At E9.5, *Dll4* exhibited high expression levels in endocardium, whereas *Dlk1* showed low expression levels (Figure S5C and S5E, upper panels). Consistent with our observations at E14.5, in E9.5 *Ets1* eKO mice, *Dlk1* mRNA levels were increased, *Dll4* mRNA levels were decreased, and N1ICD expression was reduced in endocardium when compared to littermate controls (Figure S5C through S5H).

Furthermore, by analyzing our scRNA-seq data, we found that expression levels of *Nrg1*, a Notch1 downstream target gene, and *Rbp*j, a transcriptional mediator of Notch1, were significantly reduced in endocardium of *Ets1* eKO mice at E14.5 (Figure 4L; Figure S5I). Notably, Nrg1 is crucial for cardiomyocyte proliferation and ventricular wall thickening.^13,29,30^ Taken together, these results demonstrated that loss of *Ets1* in endocardium suppressed Notch1 signaling, leading to thinning of compact myocardium.

### 3.5 Loss of *Ets1* reduces expression of *Hmcn1*, *Slit2* and *Col18a1* in coronary endothelium, leading to decreased proliferation of compact zone cardiomyocytes.

**Figure 5.**
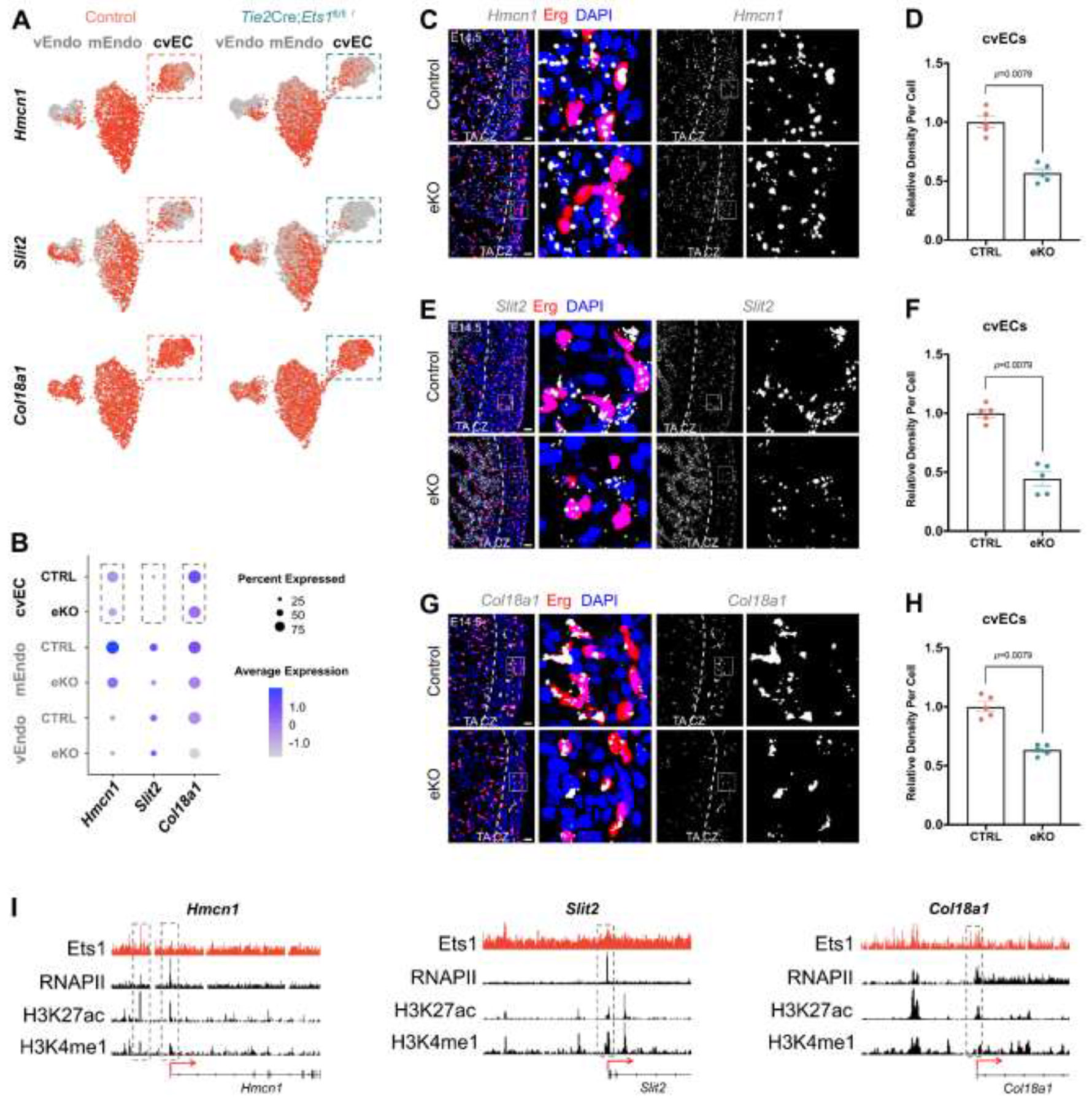
Loss of *Ets1* decreases the expression of *Hmcn1*, *Slit2* and *Col18a1*. (A and B) (A) Feature and (B) dot plots showing the expression of *Hmcn1*, *Slit2* and *Col18a1* in control and *Ets1* endothelial conditional knockout (eKO) mice. (C-H) Representative confocal images of RNAscope *in situ* hybridization (ISH) for (C) *Hmcn1*, (E) *Slit2* and (G) *Col18a1*, and quantification graphs of signal intensity for (D) *Hmcn1*, (F) *Slit2* and (H) *Col18a1* in the coronary ECs, showing the expression levels in control and *Ets1* eKO mouse hearts at E14.5 (Control, n=5; eKO, n=5, respectively). Scale bars: 20 μm. (I) Ets1 chromatin immunoprecipitation sequencing (ChIP-seq) analysis using a published dataset from human umbilical vein endothelial cells (HUVECs) showing *Hmcn1*, *Slit2* and *Col18a1* as direct targets of Ets1. cvEC, coronary vascular endothelial cell; mEndo, mural endocardial cell; vEndo, valve endocardial cells; CZ, compact zone; TA, trabecular layer. For (D), (F) and (H), data are presented as mean ± SEM and *P* values were calculated using a two-tailed Mann-Whitney test.

Next, we investigated how deletion of *Ets1* in the coronary endothelium led to thinning of the compact myocardium. Since previous studies have shown that coronary vascular ECs can promote cardiomyocyte proliferation through specific secreted factors independent of blood flow,^13,14^ we focused on proteins secreted by the coronary endothelium, particularly extracellular matrix (ECM) proteins, which are known to play a crucial role in cardiomyocyte proliferation.^31-34^ By analyzing differentially expressed genes (DEGs) in the cvEC cluster, we identified three ECM genes, *Hmcn1*, *Slit2* and *Col18a1*, which were downregulated in the coronary endothelium of *Ets1* eKO mice compared to controls (Table S1). ScRNA-seq analysis revealed that these three ECM genes were downregulated in all three types of cardiac endothelium in *Ets1* eKO mice (Figure 5A and 5B; Figure S6A). To further evaluate expression levels of these three ECM genes in the coronary endothelium, RNAscope ISH was performed at E14.5. The mRNA levels of *Hmcn1*, *Slit2* and *Col18a1* were reduced in coronary vascular ECs of *Ets1* eKO mice compared to littermate controls (Figure 5C through 5H). To determine whether these ECM genes were directly regulated by Ets1, we reanalyzed the published Ets1 ChIP-seq dataset along with available RNAPII, H3K4me3, and H3K27ac ChIP-seq data. Ets1 ChIP peaks were detected in the promoter regions of *Hmcn1*, *Slit2* and *Col18a1*, indicating that these three ECM genes were directly regulated by Ets1 (Figure 5I).

**Figure 6.**
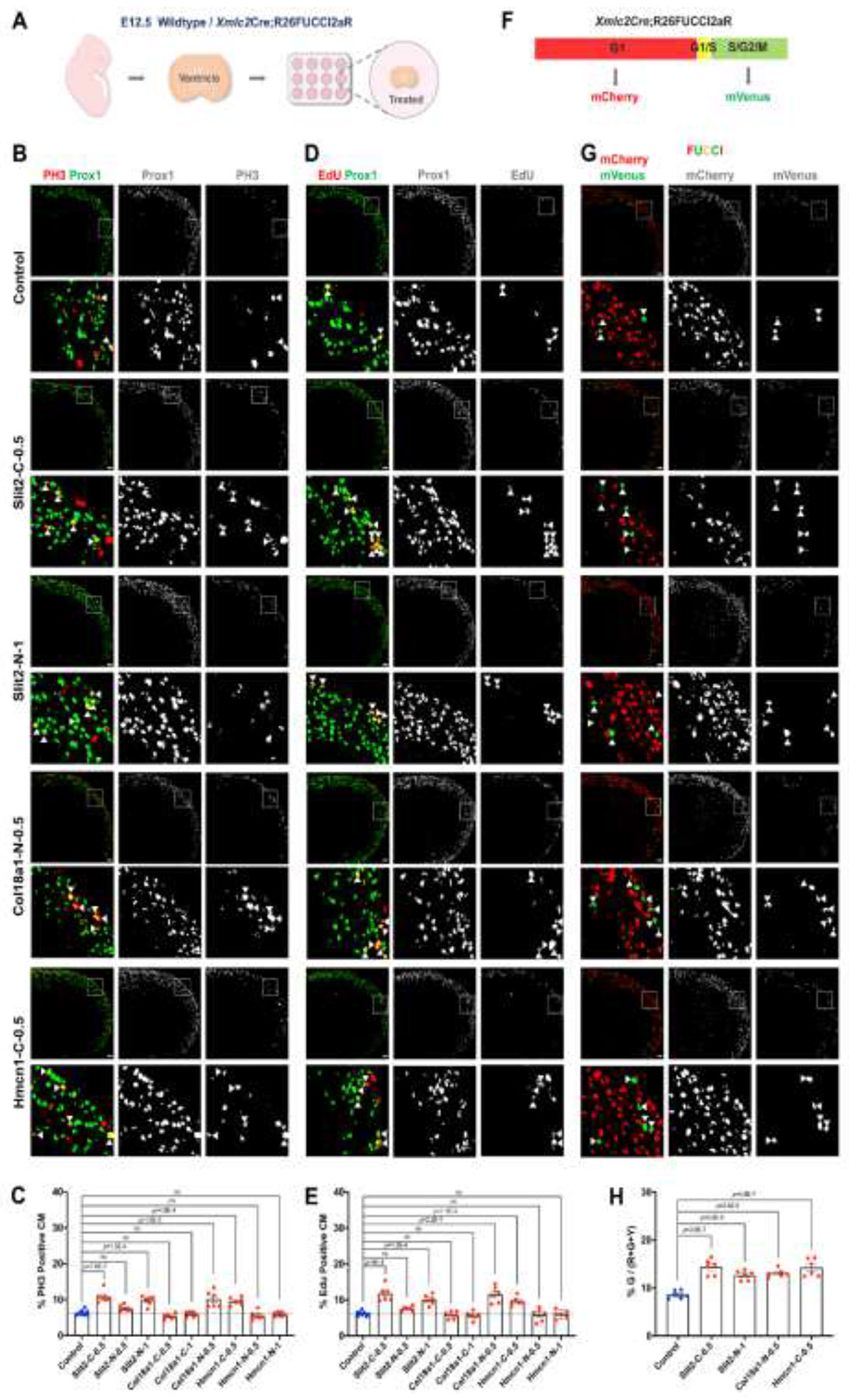
Slit2, Col18a1 and Hmcn1 promote compact zone cardiomyocyte proliferation. (A) Schematic illustrating the culture of E12.5 wildtype or *Xmlc2*Cre;R26FUCCI2aR ventricles. (B) Representative confocal images of PH3 immunofluorescence showing proliferating cardiomyocytes (white triangles) in control and recombinant protein treated-ventricles. Scale bars: 50 μm. (C) Graph of quantification of PH3 positive cardiomyocytes in control and recombinant protein treated-ventricles (Control, n=8; Treated, n=6 each). (D) Representative confocal images of EdU immunofluorescence showing proliferating cardiomyocytes (white triangles) in control and recombinant protein treated-ventricles. Scale bars: 50 μm. (E) Graph of quantification of EdU positive cardiomyocytes in control and recombinant protein treated-ventricles (Control, n=8; Treated, n=6 each). (F) Schematic illustrating that *Xmlc2*Cre;R26FUCCI2aR mice enable cardiomyocyte-specific expression of the FUCCI reporter, using the dynamic degradation of mVenus-hGem and mCherry-hCdt1 to distinguish different cell cycle stages: G1 (red), G1/S (yellow), and S/G2/M (green). (G) Representative confocal images of mCherry and mVenus immunofluorescence showing cardiomyocytes at different cell cycle stages (S/G2/M: white triangles) in control and recombinant protein treated-ventricles. Scale bars: 50 μm. (H) Graph showing the quantification of the percentage of cardiomyocytes in S/G2/M relative to the total number of cardiomyocytes in G1, G1/S, and S/G2/M in control and recombinant protein treated-ventricles (Control, n=6; Treated, n=6 each). C, C-terminal; N, N-terminal; 0.5, 0.5 μg/ml; 1, 1 μg/ml. For (C), (E) and (H), data are presented as mean ± SEM and *P* values were calculated using one-way ANOVA followed by Tukey’s multiple comparisons test.

Next, to investigate effects of the three ECM proteins on cardiomyocyte proliferation, we used a mouse embryonic heart tissue explant culture model. Ventricles from E12.5 wild-type C57BL/6 mouse hearts were cultured in a 12-well plate for 24 hours, followed by treatment with individual recombinant proteins of the three ECM genes for an additional 24 hours (Figure 6A). Given the large molecular sizes of the three ECM proteins, recombinant C-terminal and N-terminal proteins of the three ECM proteins (named Slit2-C, Slit2-N, Col18a1-C, Col18a1-N, Hmcn1-C and Hmcn1-N (Figure S6B)) were individually added at 0.5 μg/ml. If no effect was observed at this concentration, a dose of 1 μg/ml was then used. Cardiomyocyte proliferation was assessed by measuring the percentage of phospho-Histone H3 (PH3) positive cardiomyocytes or EdU positive cardiomyocytes. Cardiomyocyte nuclei were identified by *Prox1*, which has been established as a marker for cardiomyocytes at this stage.^13^

Compared to controls, treatment with Slit2-C, Col18a1-N or Hmcn1-C at 0.5 μg/ml increased the percentage of PH3 positive cardiomyocytes, whereas treatment with Slit2-N, Col18a1-C or Hmcn1-N at the same concentration had no effect (Figure 6B and 6C; Figure S7A). Slit2-N, Col18a1-C and Hmcn1-N were then tested at 1 μg/ml. At this concentration, Slit2-N increased the percentage of PH3 positive cardiomyocytes compared to controls, while Col18a1-C or Hmcn1-N still showed no effect (Figure 6B and 6C; Figure S7A). We next measured the percentage of EdU positive cardiomyocytes. Consistent with PH3 results, treatment with Slit2-C, Col18a1-N or Hmcn1-C at 0.5 μg/ml increased the percentage of EdU positive cardiomyocytes compared to controls, whereas treatment with Slit2-N, Col18a1-C or Hmcn1-N at the same concentration had no effect (Figure 6D and 6E; Figure S7B). At 1 μg/ml, Slit2-N treatment increased the percentage of EdU positive cardiomyocytes compared to controls, while Col18a1-C and Hmcn1-N still showed no effect at this concentration (Figure 6D and 6E; Figure S7B).

In addition, the Cre-responsive Fluorescent Ubiquitination Cell Cycle Indicator (FUCCI) reporter mice (R26FUCCI2aR) were crossed with cardiomyocyte-specific *Xmlc2*Cre mice to visualize cell cycle stages specifically in cardiomyocytes. *Xmlc2*Cre;R26FUCCI2aR mice enable cardiomyocyte-specific expression of the FUCCI reporter, using the dynamic degradation of mVenus-hGem and mCherry-hCdt1 to distinguish different cell cycle stages: G1 (red), G1/S (yellow), and S/G2/M (green) (Figure 6F).^35,36^ As described above, ventricles from E12.5 *Xmlc2*Cre;R26FUCCI2aR mouse hearts were cultured for 24 hours, followed by treatment with Slit2-C, Col18a1-N or Hmcn1-C at 0.5 μg/ml or Slit2-N at 1 μg/ml for an additional 24 hours to further confirm of effects of these ECM proteins on cardiomyocyte proliferation (Figure 6A). Cardiomyocyte proliferation was assessed by calculating the percentage of cardiomyocytes in S/G2/M (green) relative to the total number of cardiomyocytes in G1 (red), G1/S (yellow), and S/G2/M (green). Compared to controls, treatment with Slit2-C, Col18a1-N or Hmcn1-C at 0.5 μg/ml, or Slit2-N at 1 μg/ml, increased the percentage of cardiomyocytes in S/G2/M (green) (Figure 6G and 6H). These results demonstrated that treatment of Slit2, Col18a1 or Hmcn1 increased the proliferation of cardiomyocytes in ventricles of the normal heart. Taken together, these results indicated that Hmcn1, Slit2 and Col18a1 secreted by coronary endothelium promoted cardiomyocyte proliferation, and that their decreased expression in coronary endothelium contributed to thinning of compact zone.

### 3.6 Treatment with Slit2, Col18a1, Hmcn1 or Nrg1 restores the reduced proliferation of compact myocardium in non-compacted ventricles.

We have demonstrated that treatment with Slit2, Col18a1 or Hmcn1 promotes cardiomyocyte proliferation in ventricles of normal heart, while other studies have shown that Nrg1 is crucial for ventricular cardiomyocyte proliferation.^13,29,30^ Therefore, we assessed the effects of these three ECM proteins and Nrg1 on cardiomyocyte proliferation in the non-compacted ventricles of *Ets1* eKO mice, which have been shown to exhibit reduced proliferation.

**Figure 7.**
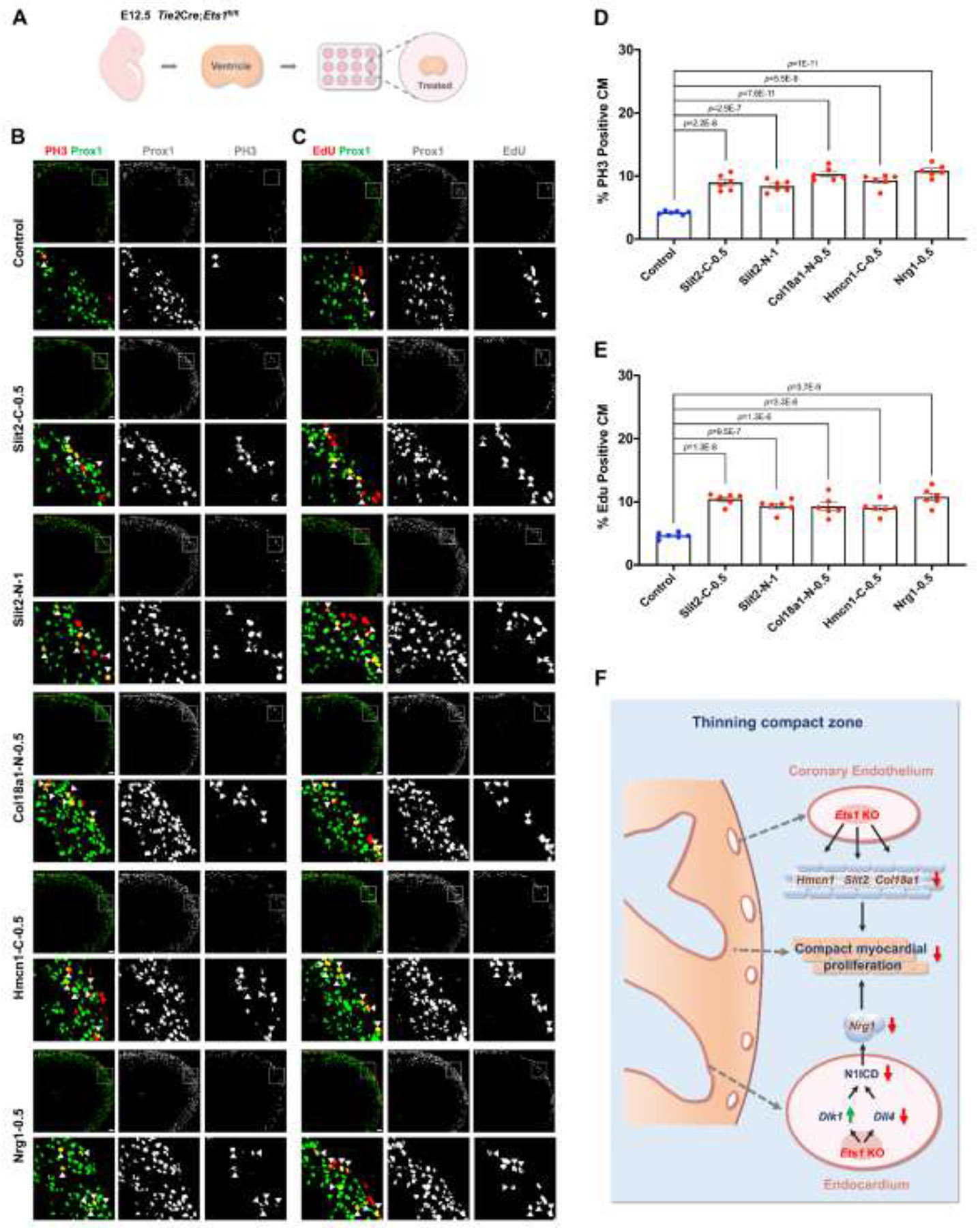
Repair effects of the identified proteins on compact zone cardiomyocyte proliferation in the ventricles of *Ets1* eKO mice. (A) Schematic illustrating the culture of E12.5 *Ets1* endothelial conditional knockout (eKO) ventricles. (B) Representative confocal images of PH3 immunofluorescence showing proliferating cardiomyocytes (white triangles) in control and recombinant protein treated-*Ets1* eKO ventricles. Scale bars: 50 μm. (C) Representative confocal images of EdU immunofluorescence showing proliferating cardiomyocytes (white triangles) in control and recombinant protein treated-ventricles. Scale bars: 50 μm. (D) Graph of quantification of PH3 positive cardiomyocytes in control and recombinant protein treated-*Ets1* eKO ventricles (Control, n=6; Treated, n=6 each). (E) Graph of quantification of EdU positive cardiomyocytes in control and recombinant protein treated-ventricles (Control, n=6; Treated, n=6 each). (F) Model depicting how *Ets1* deficiency in either endocardium or coronary endothelium leads to the thinning of the compact myocardium. Loss of *Ets1* in the endocardium inhibits the activity of the Notch1 signaling pathway by upregulating the expression of *Dlk1* and downregulating the expression of *Dll4*, thereby leading to the decreased compact zone cardiomyocyte proliferation. Loss of *Ets1* in the coronary endothelium reduces the expression of *Hmcn1*, *Slit2*, and *Col18a1*, three extracellular matrix (ECM) components, which in turn decreases the proliferation of compact zone cardiomyocytes. For (D) and (E), data are presented as mean ± SEM and *P* values were calculated using one-way ANOVA followed by Tukey’s multiple comparisons test.

A *Tie2*Cre driver line was used to delete *Ets1* in all cardiac endothelial cells. Ventricles from E12.5 *Tie2*Cre;*Ets1*^fl/fl^ (*Ets1* eKO) mouse hearts were cultured in a 12-well plate for 24 hours, followed by treatment with Slit2-C, Col18a1-N, Hmcn1-C or Nrg1 individually at 0.5 μg/ml, or Slit2-N at 1 μg/ml, or a vehicle control, for an additional 24 hours (Figure 7A). Cardiomyocyte proliferation was assessed by measuring the percentage of PH3 positive cardiomyocytes or EdU positive cardiomyocytes. Compared to controls, treatment with Slit2-C, Col18a1-N, Hmcn1-C or Nrg1 at 0.5 μg/ml, or Slit2-N at 1 μg/ml, increased the percentage of PH3 positive cardiomyocytes (Figure 7B and 7D) and EdU positive cardiomyocytes (Figure 7C and 7E) in ventricles of *Ets1* eKO mice. These results demonstrate the reparative effects of Slit2, Col18a1, Hmcn1 and Nrg1 on cardiomyocyte proliferation in ventricles with ventricular non-compaction.

## 4. Discussion

Ventricular non-compaction has been observed in JBS. Studies in both humans and mice suggest that the loss of *Ets1* is responsible for CHDs in JBS.^19,37^ In our previous study, we demonstrated that endothelial-specific deletion of *Ets1* in mice leads to a ventricular non-compaction phenotype, accompanied by reduced proliferation of compact zone cardiomyocytes.^19^ However, the specific contributions of coronary endothelium and endocardium to this thinning remained unclear. In this study, we address this by dissecting their distinct roles (Figure 7F). Our findings revealed that *Ets1* deficiency in either endocardium or coronary endothelium led to thinning of compact myocardium and uncovered the underlying molecular mechanisms. These insights enhance our understanding of cardiac endothelial regulation of compact myocardial grow and highlight potential targets for therapeutic intervention in ventricular non-compaction.

ScRNA-seq on E14.5 *Ets1* eKO and control ventricles was performed to investigate factors involved in regulating compact zone cardiomyocyte proliferation by coronary endothelium and endocardium. We first performed a comprehensive analysis of the transcriptome and cell states of cardiomyocytes and cardiac endothelial cells in ventricular non-compaction caused by endothelial gene defects. We found significant changes in the energy metabolism of endocardial cells and coronary ECs in ventricular non-compaction caused by *Ets1* eKO. This may be due to hypoxia resulting from coronary vascular defects we previously identified in *Ets1* eKO.^19^ Moreover, we found that *Ets1* eKO led to a decreased number of coronary artery ECs and coronary vein ECs in the ventricles, demonstrating defects in coronary vascular development. Notably, we identified a specific population of proliferative endocardial cells in non-compacted ventricles. Interestingly, a previous study also reported a specific population of proliferative endocardial cells in non-compacted ventricles caused by cardiac endothelial defects.^13^ Activated Notch1 signaling is associated with inhibition of endothelial cell proliferation,^38,39^ indicating that the impaired endocardial Notch1 signaling we observed in *Ets1* eKO may be the cause of these highly proliferative endocardial cells. In addition, we identified a population of immature cardiomyocytes in control ventricles at E14.5; however, these cardiomyocytes exhibited early-onset maturation in non-compacted ventricles. Future studies will also focus on identifying the location of these cardiomyocytes and highly proliferative endocardial cells, and assessing their contribution to ventricular non-compaction. Furthermore, we observed a greater number of *Hey2*^hi^ and *Bmp10*^−^ / *Bmp10*^lo^ proximal trabecular cardiomyocytes, also known as intermediate myocardium cardiomyocytes, in non-compacted ventricles. Given that we previously determined that *Ets1* eKO led to reduced cardiomyocyte proliferation in the compact zone,^19^ this is consistent with previous findings^10^ and suggests that decreased cardiomyocyte proliferation impairs compact zone expansion, thereby hindering the compaction of the proximal trabecular layer.

Proliferation of the compact zone myocardium plays a crucial role in progressive thickening of the ventricular wall. This involves both growth of the compact zone myocardium and its expansion during late embryonic stages, which facilitates integration of the trabecular myocardium.^9,10^ Disruption of compact zone cardiomyocyte proliferation leads to the thinning of compact zone myocardium in ventricular non-compaction. We then focused on DEGs between *Ets1* eKO and control endocardial cells or coronary vascular endothelial cells to identify proteins involved in regulating compact zone cardiomyocyte proliferation. Impairment of endocardial Notch1 signaling has been reported to contribute to ventricular non-compaction.^15,40^ Analysis of our scRNA-seq data from the endocardial cell cluster revealed dysregulation of two Notch1 ligand genes, *Dlk1* and *Dll4*. Dll4 has been identified as a key Notch1 ligand that activates the Notch1 signaling pathway in the endocardium.^9^ However, the role of Dlk1 in Notch1 signaling remains uncertain, particularly in ventricular development. We found that Dlk1 acts as an inhibitor of Notch1 signaling in the endocardium. *Ets1* eKO significantly reduced Notch1 activity by upregulating *Dlk1* expression and downregulating *Dll4* expression. Moreover, by analyzing our scRNA-seq data, we found that expression levels of *Nrg1*, a Notch1 downstream target known to be crucial for cardiomyocyte proliferation and ventricular wall thickening,^13,29,30^ was significantly reduced in endocardium of *Ets1* eKO mice. These results demonstrated that loss of *Ets1* in endocardium suppressed Notch1 signaling, resulting in thinning of compact myocardium. In addition, Notch1 signaling promotes ECM degradation in the trabecular layer.^3^ Cardiac trabeculation involves dynamic ECM remodeling, with ECM bubbles (ECM-rich areas surrounding trabecular myocardium) progressively reduced from E9.5 (when trabecular extension begins) to E14.5 (when it terminates).^3^ Notch1 signaling defects lead to excessive ECM accumulation in ECM bubbles and excessive trabecular growth.^3^ This is also consistent with our previous observations, where we found increased ECM protein deposition between endocardium and trabecular myocardium, as well as increased trabecular cardiomyocyte proliferation in *Ets1* eKO mice.^19^

In addition, we observed that *Dlk1* mRNA levels were increased, *Dll4* mRNA levels were decreased, and N1ICD expression was reduced in the endocardium of E9.5 *Ets1* eKO mice. By reanalyzing a published scRNA-seq dataset^41^, we examined the dynamic expression patterns of *Notch1*, *Dlk1*, and *Dll4* in nascent mesoderm, mixed mesoderm/allantois, and developing endothelium/endocardium. *Notch1* and *Dll4* were barely expressed in nascent or mixed mesoderm but were highly expressed in the endothelium/endocardium starting at E7.75, whereas *Dlk1* exhibited the opposite pattern, being highly expressed in mesodermal populations but nearly absent in endothelium/endocardium. These observations suggest that Notch1 signaling is not required for differentiation of cardiac progenitors into endothelium/endocardium, while Dlk1 may prevent excessive Notch1 activation during this process. After differentiation is complete, the Dll4-Notch1 pathway likely mediates key endothelial/endocardial functions. Interestingly, *Ets1* expression also increases in the endothelium/endocardium from E7.75, promoting *Dll4* while repressing *Dlk1*, thereby facilitating activation of the Dll4-Notch1 signaling pathway.

Coronary vascular ECs can promote cardiomyocyte proliferation through specific secreted factors, and ECM proteins play a crucial role in this process.^13,31-34^ Through our scRNA-seq analysis, we identified three ECM genes, *Hmcn1*, *Slit2* and *Col18a1*, which were expressed in the coronary endothelium and downregulated in *Ets1* eKO mice. Hmcn1 has been reported to localize to the cleavage furrow and is required for cytokinesis in mouse embryos.^42^ Slit2 has been shown to promote postnatal cardiomyocyte cytokinesis, and RhoA, a small GTPase essential for cytokinesis, is implicated in this process.^43,44^ Col18a1 has been identified as a regulator of Egfr/Erbb receptor signaling pathway and has been shown to interact with Egfr, Her2, and Itga6 to promote cancer cell proliferation.^45^ We have demonstrated, for the first time, that treatment with the Slit2, Col18a1 or Hmcn1 individual recombinant proteins increased proliferation of cardiomyocytes in the ventricles of a normal embryonic heart *ex vivo*. These results indicated that loss of *Ets1* reduced expression of *Slit2*, *Col18a1* and *Hmcn1*, three ECM genes in coronary endothelium, contributing to a thinner compact zone. Taken together, our findings demonstrate that both coronary endothelial and endocardial Ets1 are essential for regulating ventricular wall thickening.

Ventricular non-compaction can result in heart failure and, in severe cases, sudden cardiac death.^46^ Increasing evidence suggests that a thinner compact myocardium may be an independent risk factor for adverse clinical outcomes.^1,7,8^ We found that this phenotype was associated with reduced survival in *Ets1* eKO embryos, with the hearts of dead eKO embryos showing severe thinning of the compact zone.^19^ Developing methods to repair the thinning of compact myocardium in ventricular non-compaction will contribute to mitigating the pathological nature of this condition and its associated lethality. We used the thinner compact zone caused by *Ets1* eKO as a disease model to examine the effects of the three identified ECM proteins and the Notch1 downstream target Nrg1 on proliferation of *Ets1* eKO ventricular compact cardiomyocytes. Treatment with Slit2, Col18a1, Hmcn1 or Nrg1 recombinant proteins increased compact cardiomyocyte proliferation in the ventricles of *Ets1* eKO mice, demonstrating their potential to restore compact cardiomyocyte growth in the thinned compact zone. Future studies will investigate the specific mechanisms by which these three ECM genes regulate cardiomyocyte proliferation.

In our previous study, we found that *Ets1* eKO decreased the levels of *Alk1*, *Cldn5*, *Sox18*, *Robo4*, *Esm1*, and *Kdr* at E12.5 and E13.5, leading to defects in coronary vasculature development.^19^ In the current study, analysis of the scRNA-seq data from the cvEC cluster revealed that the expression of all six angiogenesis-related genes remained downregulated in the coronary endothelium of *Ets1* eKO mice compared with controls at E14.5. We also observed that the expression levels of *Alk1*, *Robo4*, and *Esm1* were extremely low at this stage, suggesting that different angiogenesis-related genes may play stage-specific roles in regulating coronary vasculature development during heart development. In addition, we identified three extracellular matrix-related genes, *Hmcn1*, *Slit2*, and *Col18a1*, that were downregulated in the coronary endothelium of *Ets1* eKO mice at E14.5, and found that *Ets1* deficiency upregulated *Dlk1* and downregulated *Dll4* in the endocardium. However, our previous bulk RNA-seq data at E12.5 only detected changes in *Dlk1*, while changes in the other genes were not evident. This discrepancy is likely due to two factors: first, bulk RNA-seq measures the average expression of mixed cardiac cell populations, which may mask changes specific to the coronary endothelium or endocardium; and second, the expression of these genes is dynamically regulated by different signals at different stages of heart development.

In summary, we performed a comprehensive transcriptomic analysis of the cardiomyocytes and cardiac endothelial cells involved in ventricular non-compaction resulting from endothelial gene defects. This provides valuable insights into cellular state alterations in ventricular non-compaction caused by endothelial gene defects. We also identified several proteins that promote proliferation of compact zone cardiomyocytes. We will further investigate precise molecular mechanisms by which these factors regulate cardiomyocyte proliferation, performing comprehensive functional analyses using genetic and pharmacological approaches, and assessing their therapeutic potential in ventricular non-compaction models. These efforts will ultimately contribute to the development of targeted therapies for ventricular non-compaction and potentially other disease processes involving cardiomyocyte proliferation.

### Limitations

Our rescue assays were designed to evaluate cardiomyocyte proliferation in the compact zone using an *ex vivo* embryonic ventricle explant model, which lacks internal structures such as the trabecular layer and septum. As a result, we were unable to assess the effects of individual recombinant proteins on the maturation and differentiation of specific cardiomyocyte subclusters. Future *in vivo* studies are planned to address this limitation and more comprehensively elucidate the mechanisms by which these proteins influence the broader spectrum of the *Ets1* mutant phenotype. In addition, due to the limitations of the *ex vivo* embryonic ventricle explant model treated with recombinant proteins, we were unable to validate the transcriptomic changes identified in our single-cell analysis using this system. Future *in vivo* studies will evaluate whether the transcriptomic changes in cardiomyocyte subclusters are rescued along with structural improvements. Overall, *in vivo* delivery and validation would reinforce further the translational relevance and mechanistic conclusions.

## Supplementary Material

Supplementary material

Table S1

Supplementary material is available at *Cardiovascular Research* online.

## Data Availability

The data underlying this article are available in the article and in its online supplementary material. ScRNA-seq data generated in this study have been deposited in the NCBI Gene Expression Omnibus (GEO) under accession numbers GSE294340 and are publicly available as of the date of publication.

## Translational Perspective

Ventricular non-compaction is a common congenital cardiomyopathy associated with heart failure and sudden cardiac death. Impaired growth of the compact myocardium is an independent risk factor for adverse clinical outcomes, underscoring the need to elucidate its underlying molecular and cellular mechanisms. Using a mouse model, we show that Ets1 regulates compact myocardium development by sustaining Notch1 signaling in the endocardium and promoting secretion of proliferative extracellular matrix (ECM) components from the coronary endothelium. Notably, treatment with these ECM proteins or the Notch1 downstream effector Nrg1 restored the impaired compact myocardial proliferation. These findings highlight the critical role of Ets1-regulated endothelial-secreted factors in compact myocardial development and suggest novel therapeutic strategies for ventricular non-compaction and potentially other conditions involving cardiac regeneration, with endothelial cells serving as a promising therapeutic entry point.
