# Supplementary material for: Ets1-regulated endothelial-secreted factors promote compact myocardial growth and contribute to the pathogenesis of ventricular non-compaction
Source: Cardiovasc Res. Author manuscript; Available in PMC 2026 Apr 17. (PMC13089644; doi:10.1093/cvr/cvaf264)
Supplement: Supplementary material [file NIHMS2154083-supplement-Supplementary_material.pdf]

SUPPLEMENTARY MATERIAL

ETS1-Regulated Endothelial-Secreted Factors Promote Compact Myocardial Growth and Contribute to the Pathogenesis of Ventricular Non-Compaction

Lu Wang[1,*,#], Zeyu Chen[2,*], Aiden Tang[1], Zhe Yu[3], Bin Zhou[4], Sylvia M. Evans[3], Ju Chen[2,#], Paul Grossfeld[1,5,#]

[1]Department of Pediatrics, UCSD School of Medicine, La Jolla, CA 92093, USA;
[2]Department of Medicine, University of California San Diego, La Jolla, CA 92093, USA;
[3]Department of Pharmacology, Skaggs School of Pharmacy and Pharmaceutical Sciences, University of California San Diego, La Jolla, CA 92093, USA;
[4]Department of Pediatrics, The University of Chicago, Chicago, IL 60637, USA;
[5]Division of Cardiology, Rady Children's Hospital, San Diego, CA 92123, USA.
*These authors contributed equally

[#]Address correspondence to:

Paul Grossfeld, Division of Cardiology, Rady Children's Hospital, 3020 Children's Way, MC5004, San Diego, CA 92123; Phone: 858-966-5855; Email: pgrossfeld@health.ucsd.edu.

Or

Ju Chen, Department of Medicine, University of California San Diego, 9500 Gilman Drive, La Jolla, CA 92093; Phone: 858-822-4276; Email: juchen@health.ucsd.edu;

Or

Lu Wang, Department of Pediatrics, UCSD School of Medicine, 9500 Gilman Drive, La Jolla, CA 92093; Phone: 858-966-5855; Email: luw059@health.ucsd.edu.

Expanded Materials & Methods

Histology

Mouse hearts were dissected in PBS and fixed overnight at 4 °C in 4% PFA. After fixation, they were dehydrated, embedded in paraffin, and sectioned into 8-μm slices using a Microtome. These sections were then stained with hematoxylin and eosin following standard protocols, mounted on slides, and imaged using a Hamamatsu NanoZoomer 2.0-HT Slide Scanner. Representative images from each group were selected to illustrate the average or median histological features of the group.

Immunofluorescence

Mouse embryos were dissected in PBS and fixed overnight at 4 °C in 4% PFA. After fixation, they were sequentially incubated in 5%, 10%, 15%, and 20% sucrose in PBS, embedded in OCT Tissue-Tek (Thermo Fisher Scientific), and sectioned into 6-μm slices using a Leica CM 3050S Cryostat (Leica Microsystems). Sections were blocked with 10% donkey serum in 0.1% PBST (PBS with 0.1% Triton-X 100) at room temperature for 1 hour, followed by overnight incubation at 4 °C with primary antibodies diluted in blocking solution. After three washes in 0.1% PBST at room temperature, sections were incubated with secondary antibodies and DAPI (1:1,000) at room temperature for 1 hour. Sections were then washed three more times in 0.1% PBST and mounted in DAKO fluorescence mounting medium (Agilent). To validate antibody specificity, an IgG isotype control from the same species was used in place

of the primary antibody, and immunofluorescence was performed as described above. To distinguish true target staining from background, secondary antibody-only controls were included, omitting the primary antibodies. Immunofluorescence images were acquired using a Zeiss LSM 880 Airy Scan Confocal Microscope. Primary and secondary antibodies used are listed in Table S2. Representative images for each group were selected to reflect the average or median fluorescence patterns of the group.

RNAscope in situ hybridization

RNAscope in situ hybridization (ISH) was performed on cryosections of mouse hearts using the RNAscope Multiplex Fluorescent Reagent Kit V2 (ACD) according to the manufacturer's instructions. For sections undergoing antibody co-staining following RNAscope ISH, subsequent steps adhered to the immunofluorescence protocol described above. Images were acquired using a Zeiss LSM 880 Airy Scan Confocal Microscope. The RNAscope probes used are listed in Table S2. Representative images for each group were selected to reflect the average or median fluorescence signal within the group.

Embryonic Ventricle Tissue Explant Culture

Embryonic ventricle tissue explant culture was performed as previously described.[13] Ventricles from E12.5 wild-type C57BL/6 mouse hearts, *Xmlc2*Cre;R26FUCCI2aR mouse hearts or *Tie2*Cre;*Ets1*$^{fl/fl}$ (*Ets1* eKO) mouse hearts were dissected and

cultured in 1:200 Matrigel (BD Biosciences) with EGM-2MV culture medium (Lonza, CC-3202) in a 12-well plate for 24 hours, followed by treatment with recombinant proteins for an additional 24 hours.

Proliferation assays

Immunofluorescence was performed as described above. Cardiomyocyte proliferation was assessed by measuring the percentage of PH3-positive cardiomyocytes or EdU-positive cardiomyocytes, or by calculating the percentage of cardiomyocytes in S/G2/M (green) relative to the total number of cardiomyocytes in G1 (red), G1/S (yellow), and S/G2/M (green). Cardiomyocyte nuclei were identified by Prox1 staining. For EdU analysis, 400 ng of EdU was added to 1 ml of culture medium 30 minutes before sample collection. EdU-positive cells were detected using Click-iT™ EdU Cell Proliferation Kit (Invitrogen, C10340) following the manufacturer's instructions. For each parameter, 3 to 6 sections were analyzed and sections from equivalent coronal planes were compared.

Whole Embryo Culture

E9 mouse embryos were dissected and cultured for 24 hours in a medium consisting of 75% fetal bovine serum (FBS) and 25% DMEM, with or without recombinant DLK1 protein. Following culture, embryos were collected for RNAscope ISH and immunofluorescence staining.

Single Cell RNA sequencing

Two control and two *Ets1* eKO samples were used for single cell RNA sequencing (scRNA-seq), with each sample consisting of three E14.5 mouse ventricles. Tissue samples were enzymatically dissociated using 0.25% trypsin (Gibco) at 37 °C for 20-30 minutes on a thermomixer and gently pipetted to obtain a single-cell suspension. The digestion reaction was quenched by adding DMEM containing 10% fetal bovine serum (FBS). Cells were then passed through a 40-μm cell strainer, centrifuged at 400 g, and resuspended in DMEM with 2% FBS. Cell viability was confirmed using Trypan Blue staining. Single-cell capture was performed using the Chromium Next GEM Single Cell 3' Reagent Kits v3.1 (10X Genomics) following the manufacturer's instructions. Briefly, single cells were partitioned into nanoliter-scale Gel Beads-in-Emulsion (GEMs) using the Chromium Next GEM Chip G in the Chromium Controller. Immediately after GEM generation, the Gel Bead was dissolved, primers were released, and co-partitioned cells were lysed, allowing their mRNA to be reverse transcribed into barcoded cDNA. The cDNA was then purified, amplified, enzymatically fragmented, and further amplified via PCR to generate sufficient material for library construction. Library preparation included end repair, A-tailing, adaptor ligation, and sample index PCR, resulting in constructs containing the sample index, UMI sequences, barcode sequences, and Illumina sequencing primers P5 and P7 at both ends. The libraries were sequenced on the Illumina NextSeq S4 platform to a depth of 140–270 million reads per sample.

scRNA-seq Analysis

The reads were aligned to the mouse mm10 transcriptome and converted to mRNA counts using Cell Ranger (v7.1.0, 10X Genomics). Count matrices were imported into Seurat (v4.3.0) and normalized using SCTransform, correcting for sequencing depth, cell cycle effects, and mitochondrial content. Genes detected in fewer than six cells were excluded. PCA was performed for dimensionality reduction, with key components selected using the Elbow method and JackStraw test. Graph-based clustering was applied, followed by UMAP for visualization. Cluster-specific marker genes were identified using FindAllMarkers with DESeq2, requiring a log-fold change > 0.25 and expression in >20% of cells in both WT and MT groups. Clusters were annotated based on canonical markers from literature and embryonic mouse heart atlases.

Cells with 200–7000 detected genes were retained, and ambient RNA contamination was corrected using CellBender. Cells with mitochondrial gene content >20%, ribosomal >35%, or hemoglobin >2% were removed to exclude debris and apoptotic cells. Scrinvex assessed read quality, filtering out cells with exon ratios <0.18 (damaged) or >0.5 (dead). Retained cells met thresholds of total reads <50,000, exonic reads <18,000, and intronic reads <25,000. DoubletFinder (v2.0.3) identified and removed doublets (score >0.4), ensuring high-quality single-cell data for analysis. Batch correction was performed using Harmony, and the data was scaled with Seurat's ScaleData function for clustering. Sample distribution and gene detection per cluster were checked to rule out batch effects.

Chromatin immunoprecipitation sequencing analysis

Human umbilical vein endothelial cell ETS1, RNAPII, H3K4me1, and H3K27ac chromatin immunoprecipitation sequencing (ChIP-seq) data were obtained from the Gene Expression Omnibus (GEO) under accession number GSE93030. ChIP-seq coverage tracks were generated from raw sequencing reads using Basepair (https://www.basepairtech.com/).

Quantification of thickness of compact zone myocardium

Quantification was performed as previously described.[19] Serial sections of embryonic hearts were prepared, and hematoxylin and eosin staining was performed to visualize ventricular structures. The thickness of the compact myocardium was measured in heart sections from equivalent planes using NDP.view 2 software. Measurements were taken at the region where the compact myocardium corresponded to the widest trabecular myocardium. For each parameter, 3 to 6 sections were analyzed, and comparisons were made between sections from equivalent coronal planes.

Table S2. List of regents used.

Antibodies	Source	Cat. No.
Rabbit mAb anti-ETS1	Cell Signaling Technology	14069S
Rat mAb anti-CD31	BD	550274
Rabbit mAb anti-ERG	Abcam	ab92513
Rabbit pAb anti-phospho-Histone H3	Sigma-Aldrich	06-570
Rabbit mAb anti-Cleaved NOTCH1	Cell Signaling Technology	4147
Goat pAb anti-PROX1	R&D Systems,	AF2727
Rabbit pAb anti-mCherry	Abcam	Ab167453
Chicken pAb anti-GFP	Abcam	Ab13970
Donkey anti-Chicken IgG, Alexa 488	Invitrogen	A78948
Donkey anti-Goat IgG, Alexa 488	Invitrogen	A32814
Donkey anti-Rabit IgG, Alexa 647	Invitrogen	A32795
Donkey anti-Rat IgG, Alexa 594	Invitrogen	A21209
**Probes**	**Source**	**Cat. No.**
RNAscope® Probe-Mm-Bmp10	ACD	415921
RNAscope® Probe-Mm-Hey2-C2	ACD	404651-C2
RNAscope® Probe - Mm-Dlk1-C2	ACD	405971-C2
RNAscope® Probe - Mm-Dll4-C2	ACD	319971-C2
RNAscope® Probe - Mm-Hmcn1	ACD	834921
RNAscope® Probe - Mm-Col18a1-C3	ACD	483801-C3
RNAscope® Probe - Mm-Slit2-C2	ACD	449691-C2

Table S3. List of primers used.

Primers	Sequence (5' -> 3')	Application
Ets1-FL-F	CTCTCATTTGCCATCTTTAGC	Genotyping
Ets1-FL-R	GTTTGTTTGTTTGTTTGTTTGTTC	Genotyping
Cre-F	CCGGGCTGCCACGACCAA	Genotyping
Cre-R	GGCGCGGCAACACCATTTTT	Genotyping
*Pdgfb*CreERT2-F	CCAGCCGCCGTCGCAACT	Genotyping
*Pdgfb*CreERT2-R	GCCGCCGGGATCACTCTCG	Genotyping
FUCCI-Rosa26-F	CTCTGCTGCCTCCTGGCTTCT	Genotyping
FUCCI-Rosa26-R	CGAGGCGGATCACAAGCAATA	Genotyping
FUCCI-mT/mG-R	TCAATGGGCGGGGGTCGTT	Genotyping

Supplemental Figures:

Figure S1

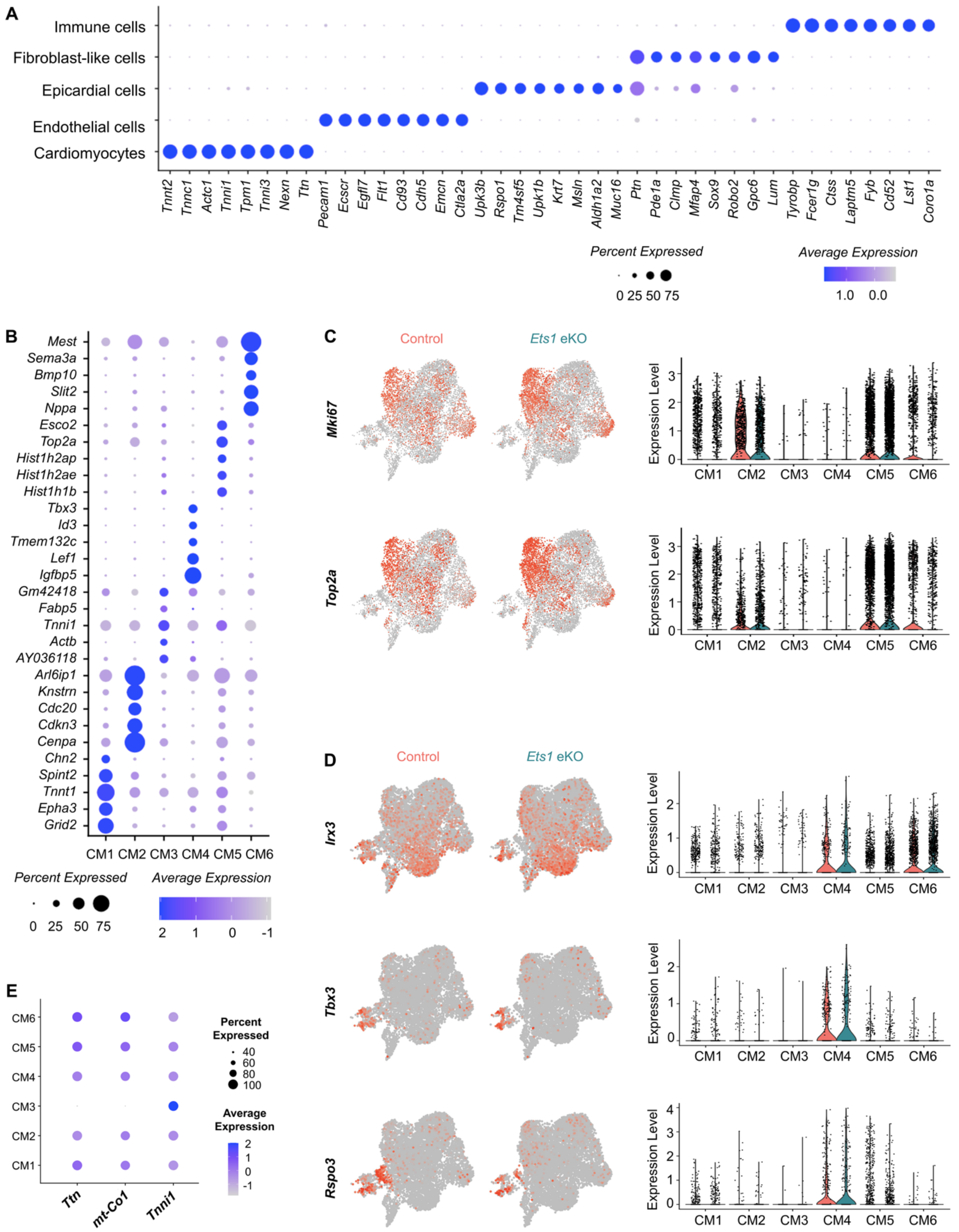

Figure S1. ***Ets1*** **endothelial deletion changes the cardiomyocyte transcriptome.** (A) Expression of the top eight genes defining each cluster. (B) Expression of the top five genes defining each cardiomyocyte subcluster. (C) Feature and violin plots displaying the expression of genes specific to proliferating cells. (D) Feature and violin plots displaying the expression of genes defining atrioventricular canal cardiomyocyte subcluster. (E) Dot plot showing the expression of *Ttn*, *mt-Co1* and *Tnni1*. CM, cardiomyocyte.

Figure S2

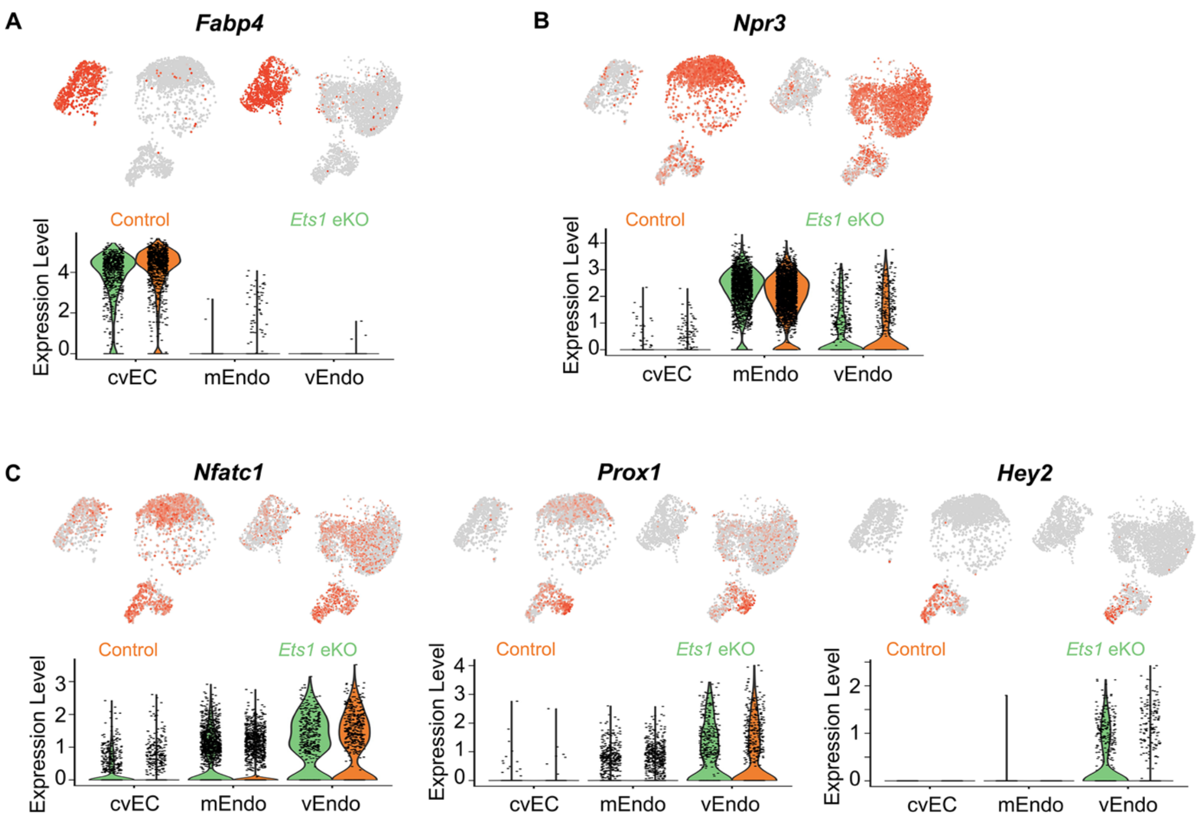

Figure S2. Expression of marker genes used to identify three cardiac endothelial subpopulations. (A-C) Feature and violin plots displaying the expression of (A) coronary vascular endothelial cell (cvEC), (B) mural endocardial cell (mEndo) and (C) valve endocardial cell (vEndo) specific genes.

Figure S3

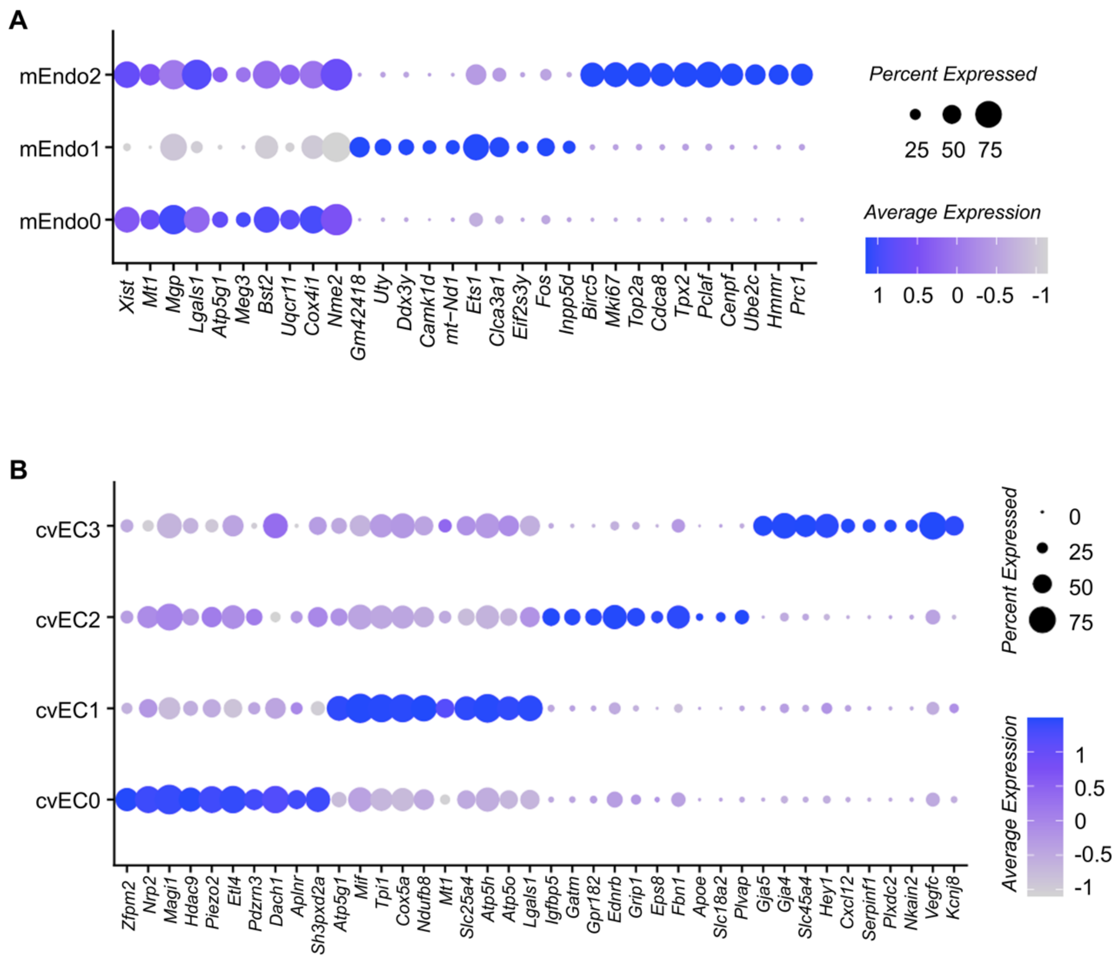

Figure S3. Expression of the top genes defining each endocardial and coronary vascular endothelial subcluster. (A) Expression of the top ten genes defining each mural endocardial cell (mEndo) subcluster. (B) Expression of the top ten genes defining each coronary vascular endothelial cell (cvEC) subcluster.

Figure S4

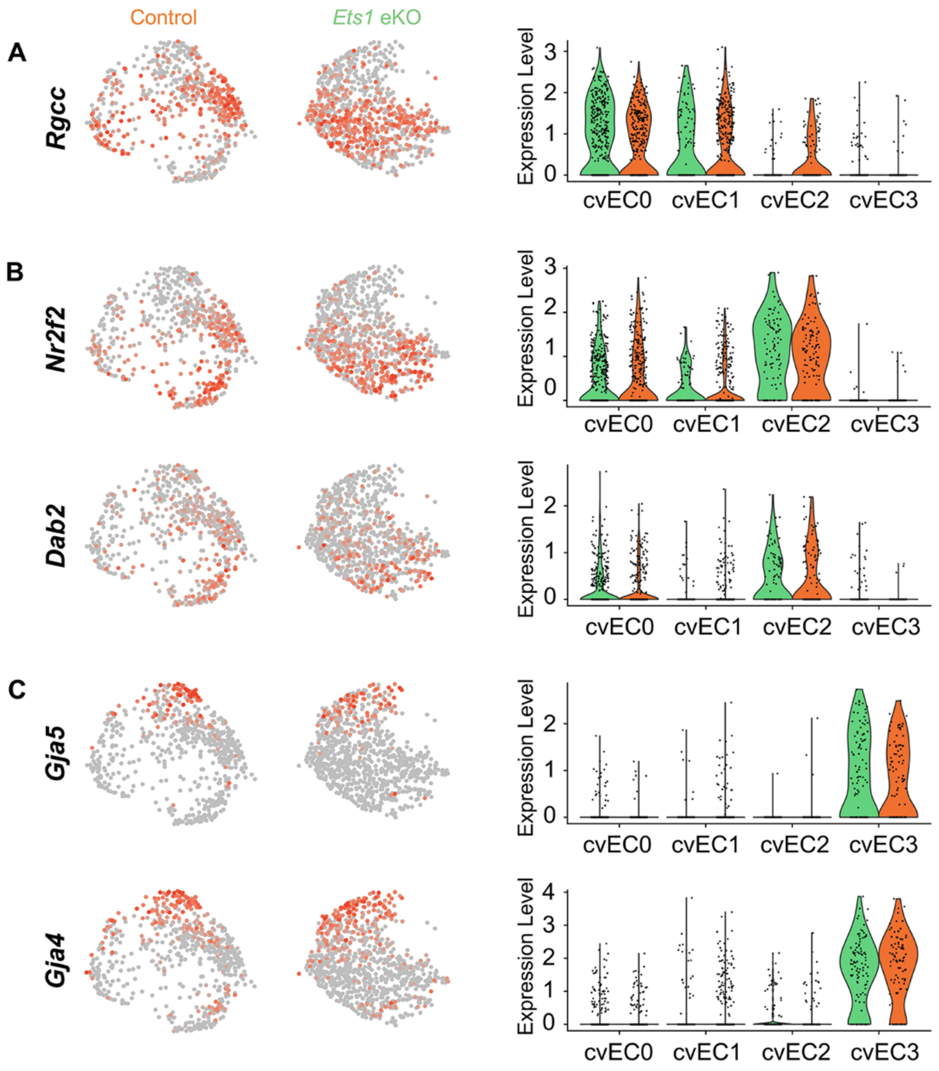

Figure S4. Expression of marker genes used to identify each coronary vascular endothelial subcluster. (A-C) Feature and violin plots displaying the expression of (A) capillary endothelial cell (EC), (B) venous EC and (C) arterial EC specific genes. cvEC, coronary vascular endothelial cell.

Figure S5

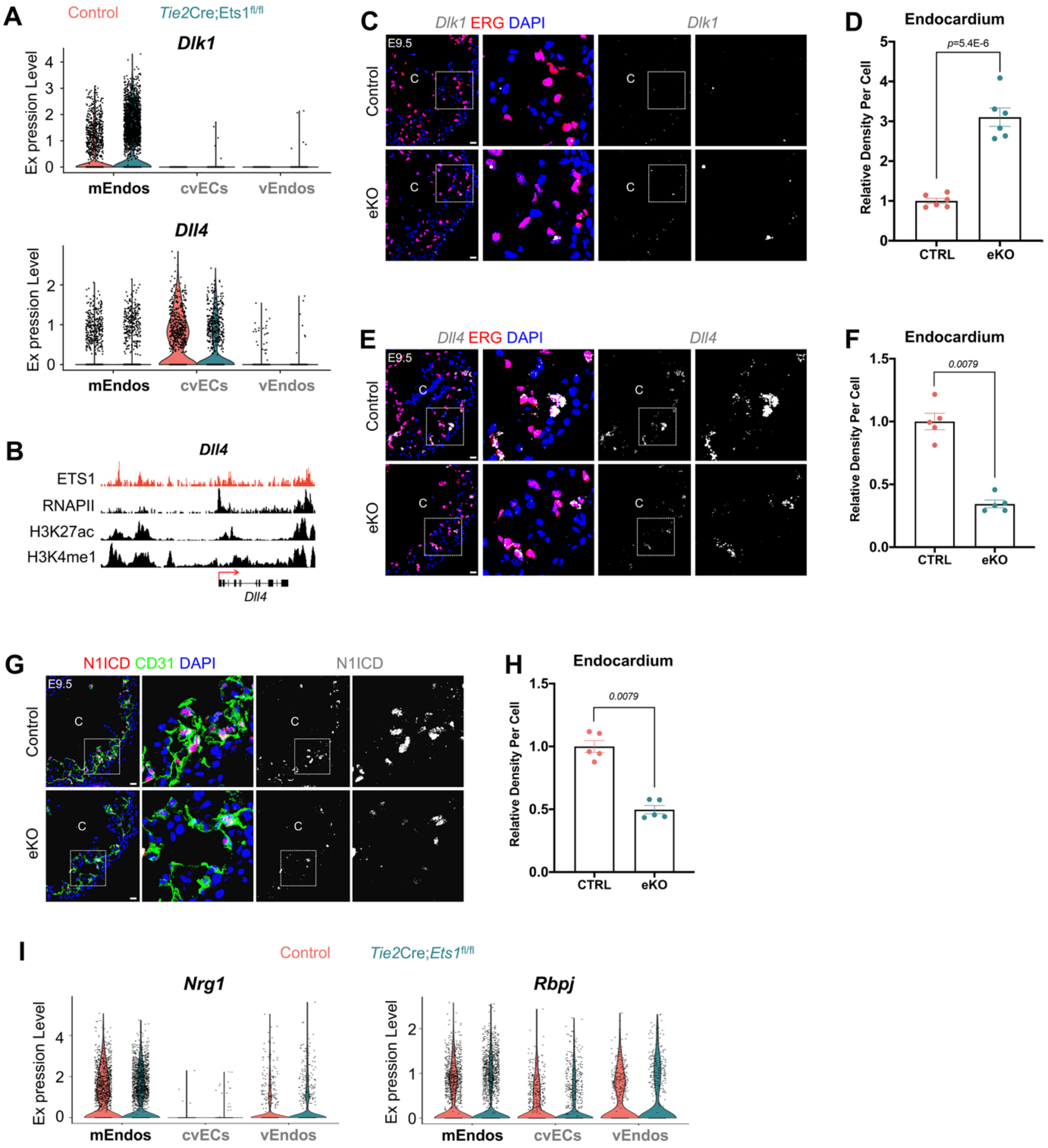

Figure S5. Loss of *Ets1* in the endocardium suppresses the NOTCH1 signaling pathway. (A) Violin plots showing the expression of *Dlk1* and *Dll4*. (B) ETS1 chromatin immunoprecipitation sequencing (ChIP-seq) analysis using a published dataset from

human umbilical vein endothelial cells (HUVECs) showing *Dll4* as a direct target gene of ETS1. (C-F) Representative confocal images of RNAscope *in situ* hybridization (ISH) for (C) *Dlk1* and (E) *Dll4*, and quantification graphs of signal intensity for (D) *Dlk1* and (F) *Dll4* in the endocardium, showing the expression levels in control and *Ets1* endothelial conditional knockout (eKO) mouse hearts at E9.5 (Control, n=6; eKO, n=6 for *Dlk1*; Control, n=5; eKO, n=5 for *Dll4*). Scale bars: 20 μm. (G and H) (G) Representative confocal images of NOTCH1 intracellular domain (N1ICD) immunofluorescence and (H) quantification graph of signal intensity for N1ICD in the endocardium, showing the expression levels in control and *Ets1* eKO mouse hearts at E9.5 (Control, n=5; eKO, n=5). Scale bars: 20 μm. (I) Violin plots showing the expression of *Nrg1* and *Rbpj*. cvEC, coronary vascular endothelial cell; mEndo, mural endocardial cell; vEndo, valve endocardial cells; C, chamber.

Figure S6

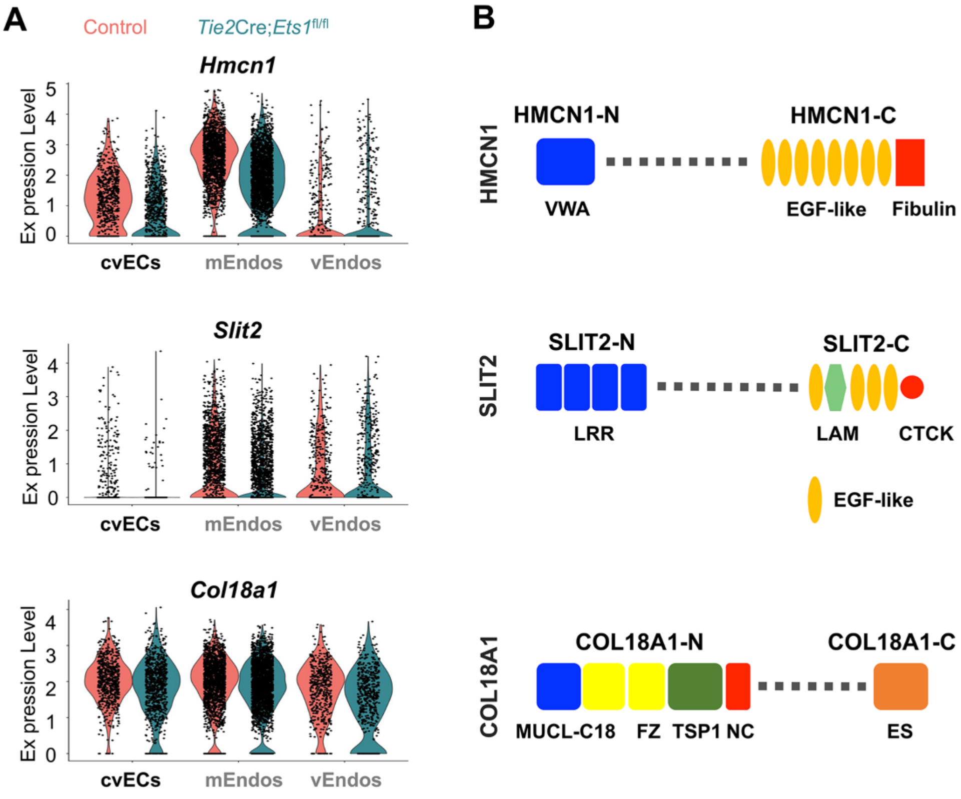

Figure S6. Loss of *Ets1* decreases the expression of *Hmcn1*, *Slit2* and *Col18a1*. (A) Violin plots showing the expression of *Hmcn1*, *Slit2* and *Col18a1* in control and *Ets1* endothelial conditional knockout (eKO) mice. cvEC, coronary vascular endothelial cell; mEndo, mural endocardial cell; vEndo, valve endocardial cells. (B) Domain organization of the recombinant C-terminal and N-terminal proteins used. VWA, von Willebrand A; LRR, leucine-rich repeat; LAM, Laminin G-like; MUCL-C18, mucin-like domain in ColXVIII; FZ, frizzled domain; TSP1, thrombospondin 1; ES, endostatin.

Figure S7

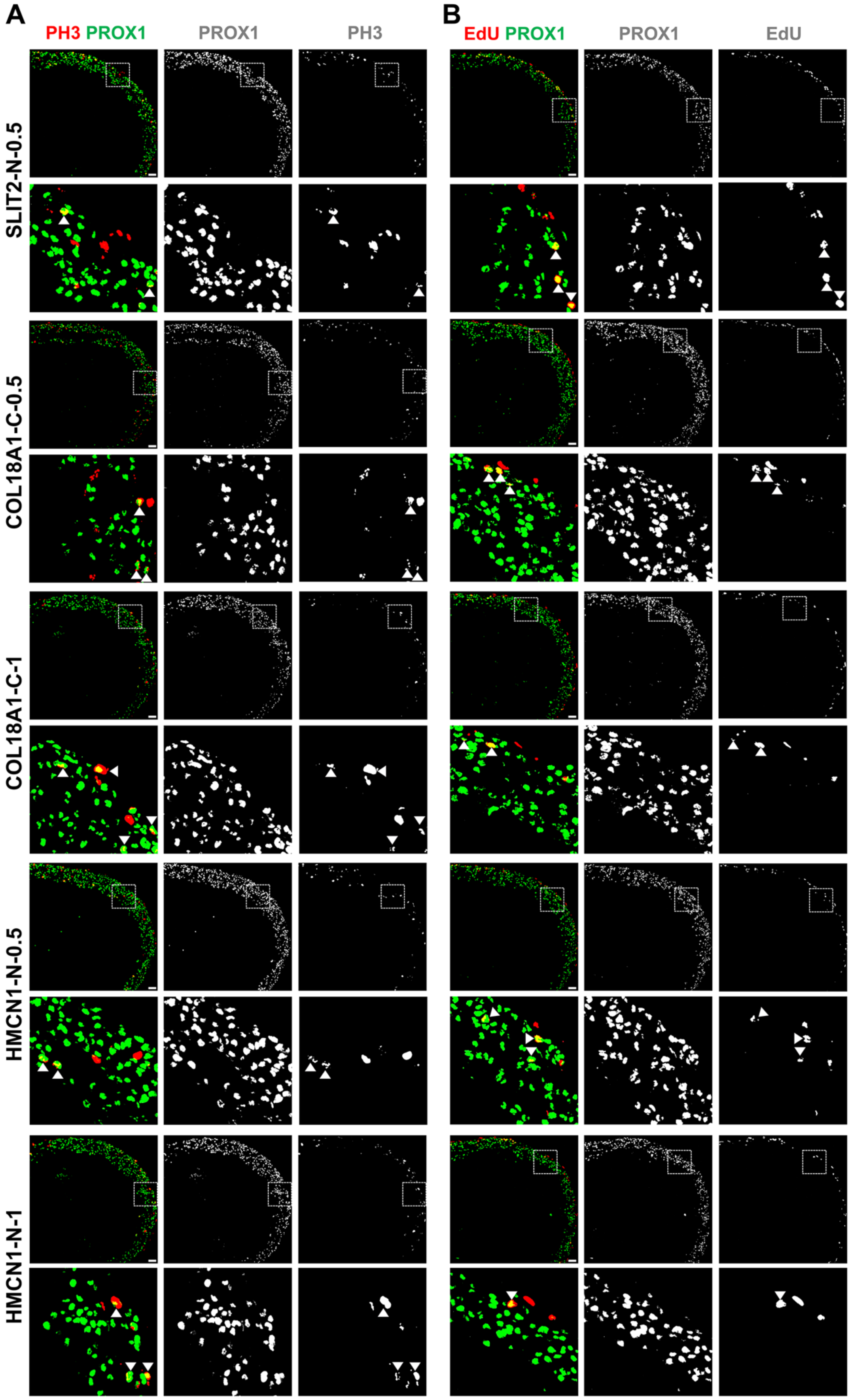
A
PH3 PROX1
PROX1
PH3
B
EdU PROX1
PROX1
EdU
SLIT2-N-0.5
COL18A1-C-0.5
COL18A1-C-1
HMCN1-N-0.5
HMCN1-N-1

Figure S7. SLIT2, COL18A1 and HMCN1 promote cardiomyocyte proliferation. (A) Representative confocal images of PH3 immunofluorescence showing proliferating cardiomyocytes (white triangles) in control and recombinant protein treated-ventricles. Scale bars: 50 μm. (B) Representative confocal images of EdU immunofluorescence showing proliferating cardiomyocytes (white triangles) in control and recombinant protein treated-ventricles. Scale bars: 50 μm.
